# Enhanced accumulation of harpagide and 8-*O*-acetyl-harpagide in *Melittis melissophyllum* L. agitated shoot cultures analyzed by UPLC-MS/MS

**DOI:** 10.1371/journal.pone.0202556

**Published:** 2018-08-22

**Authors:** Ewa Skrzypczak-Pietraszek, Katarzyna Reiss, Paweł Żmudzki, Jacek Pietraszek

**Affiliations:** 1 Chair and Department of Pharmaceutical Botany, Faculty of Pharmacy, Collegium Medicum, Jagiellonian University, Kraków, Poland; 2 Chair of Pharmaceutical Chemistry, Faculty of Pharmacy, Collegium Medicum, Jagiellonian University, Kraków, Poland; 3 Department of Software Engineering and Applied Statistics, Faculty of Mechanical Engineering, Cracow University of Technology, Kraków, Poland; Fred Hutchinson Cancer Research Center, UNITED STATES

## Abstract

Harpagide and its derivatives have valuable medicinal properties, such as anti-inflammatory, analgesic and potential antirheumatic effects. There is the demand for searching plant species containing these iridoids or developing biotechnological methods to obtain the compounds. The present study investigated the effects of methyl jasmonate (MeJa, 50 μM), ethephon (Eth, 50 μM) and L-phenylalanine (L-Phe, 2.4 g/L of medium), added to previously selected variant of Murashige and Skoog medium (supplemented with plant growth regulators: 6-benzylaminopurine 1.0 mg/L, α-naphthaleneacetic acid 0.5 mg/L, gibberellic acid 0.25 mg/L) on the accumulation of harpagide and 8-*O*-acetyl-harpagide in *Melittis melissophyllum* L. agitated shoot cultures. Plant material was harvested 2 and 8 days after the supplementation. Iridoids were quantitatively analyzed by the UPLC-MS/MS method in extracts from the biomass and the culture medium. It was found that all of the variants caused an increase in the accumulation of harpagide. In the biomass harvested after 2 days, the highest harpagide content of 247.3 mg/100 g DW was found for variant F (L-Phe and Eth), and the highest 8-*O*-acetyl-harpagide content of 138 mg/100 g DW for variant E (L-Phe and MeJa). After 8 days, in some variants, a portion of the metabolites was released into the culture medium. Considering the total amount of the compounds (in the biomass and medium), the highest accumulation of harpagide, amounting to 619 mg/100 g DW, was found in variant F, and the highest amount of 8-*O*-acetyl-harpagide, of 255.4 mg/100 g DW, was found in variant H (L-Phe, MeJa, Eth) when harvested on the 8^th^ day. These amounts were, respectively, 24.7 and 4.8 times higher than in the control culture, and were, respectively, 15 and 6.7 times higher than in the leaves of the soil-grown plant. The total amount of the two iridoids was highest for variant F (0.78% DW) and variant H (0.68% DW) when harvested on the 8^th^ day. The results indicate that the agitated shoot cultures of *M*. *melissophyllum* can be a rich source of harpagide and 8-*O*-acetyl-harpagide, having a potential practical application. To the best of our knowledge we present for the first time the results of the quantitative UPLC-MS/MS analysis of harpagide and 8-*O*-acetyl-harpagide in *M*. *melissophyllum* shoot cultures and the enhancement of their accumulation by means of medium supplementation with elicitors and precursor.

## Introduction

**Fig 1:**
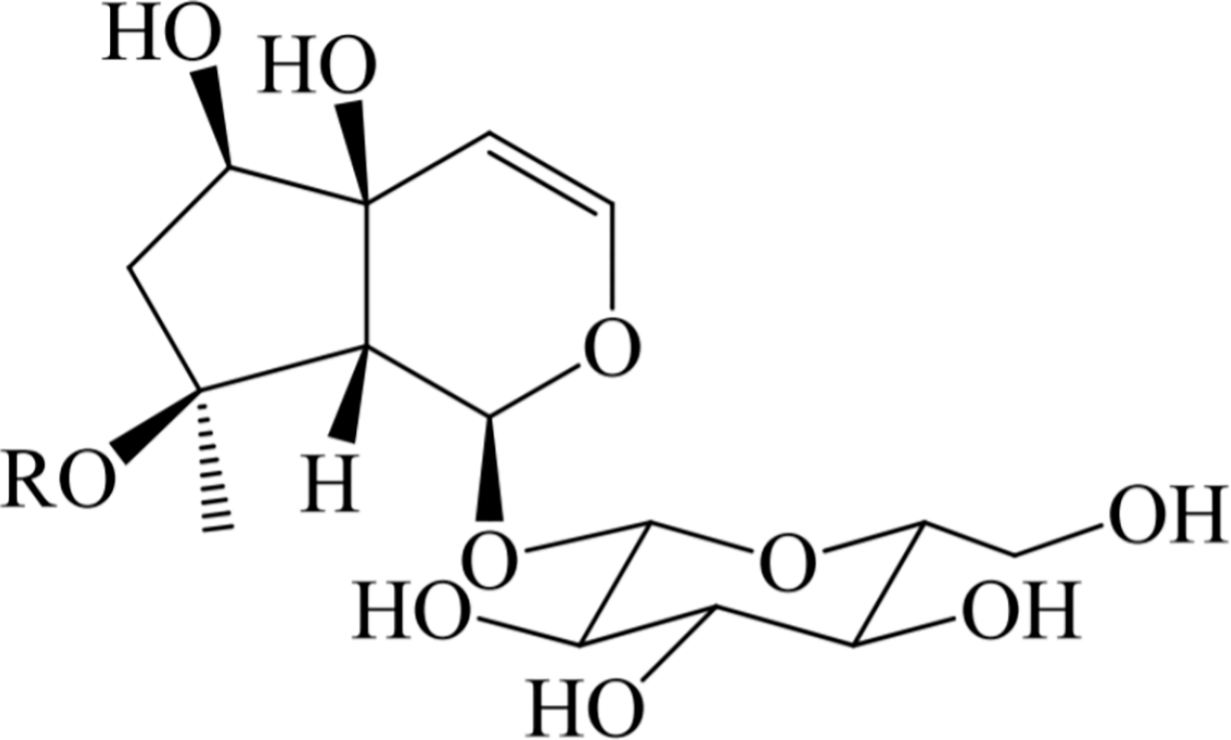
Chemical structures of harpagide and two related iridoid glycosides. Harpagide (R = H), 8-*O*-acetyl-harpagide (R = Ac) and harpagoside (R = cinnamoyl).

Harpagoside (8-*O*-cinnamoyl ester of harpagide–Fig 1) and harpagide are the major iridoid constituents of the secondary roots (tubers) of *Harpagophytum procumbens* (Burch.) DC. ex Meisn.–a medicinal plant currently listed in the European Pharmacopoeia [1]. Both compounds are responsible for such medicinal properties as anti-inflammatory, analgesic and potential anti-rheumatic effects [2]. The demand for harpagoside and harpagide containing plant medicines is high but the availability of *H*. *procumbens* roots is limited. This is the reason for searching for other species containing harpagide and its derivatives or developing biotechnological methods to obtain these compounds [3]. Plant *in vitro* culture systems can offer alternative possibilities for the therapeutically important secondary metabolite source [4–7]. Not only suspension cultures but also organ cultures (e.g. shoot and hairy or adventitious root cultures) can be considered as production systems (e.g. [5, 8–13]). It is possible to scale up plant organ cultures (up to 10-20 L) because numerous types of temporary immersion systems are developed and used (e.g. [12, 14]). Numerous biotechnological strategies, such as elicitation, the precursor feeding and others [15–20], have been developed to enhance secondary metabolite accumulation in the cultures.

The aim of the study was to investigate the influence of methyl jasmonate, ethephon and L-phenylalanine on the accumulation of harpagide and 8-*O*-acetyl-harpagide in *M*. *melissophyllum* subsp. *melissophyllum* agitated shoot cultures. To the best of our knowledge we present for the first time the results of the quantitative UPLC-MS/MS analysis of harpagide and 8-*O*-acetyl-harpagide in *M*. *melissophyllum* shoot cultures and the enhancement of their accumulation by means of medium supplementation with elicitors and precursor.

*Melittis melissophyllum* L. (Lamiaceae) is a perennial plant inhabiting woody and shady areas in Central and Southern parts of Europe. The species contains a wide variety of chemical compounds that are responsible for its different medicinal properties used in the traditional medicine such as e.g. antispasmodic, digestive, activity against nervous anxiety, insomnia, eye inflammations, sore throat and cough [21, 22].

Several studies have been conducted on *M*. *melissophyllum* chemical compounds such as flavonoids [23], phenolic acids [24], polysaccharides [25], the essential oil components (e.g. [26]), iridoids [27–29]. Recently, Venditti, Frezza [30] reassessed *M*. *melissophyllum* subsp. *melissophyllum* iridoidic fraction, isolating and identifying one new for the species compound allobetonicoside and the five previously known–harpagide (Fig 1), 8-*O*-acetyl-harpagide (Fig 1), melittoside, monomelittoside and ajugoside.

*M*. *melissophyllum* species includes three subspecies (subsp. *albida* (Guss.) P.W. Ball; subsp. *carpatica* (Klokov) P.W. Ball; subsp. *melissophyllum* L.), which differ in some morphological characteristics and occurrence area. *M*. *melissophyllum* subsp. *melissophyllum* grows, among others, in Poland. Italian researchers found that subsp. *melissophyllum* and subsp. *albida* differ in chemical composition of the essential oil [31] and iridoid fraction [32]. *M*. *melissophyllum* subsp. *albida*, apart from harpagide, 8-*O*-acetyl-harpagide, melittoside and allobetonicoside also contains such iridoids as virginioside and geniposidic acid, unknown for subsp. *melissophyllum*.

## Materials and methods

### Plant material

Seeds and aerial parts of *M*. *melissophyllum* subsp. *melissophyllum* were obtained from Polish Botanical Gardens (Poznań, Warszawa) in 2012 and 2015, respectively. The plant identity was verified by Dr Ewa Skrzypczak-Pietraszek, referring to available literature [33]. Voucher specimens were deposited (Department of Pharmaceutical Botany, Jagiellonian University, Collegium Medicum, Kraków, Poland; No M.m.2012.03, M.m.2015.01). Aerial parts of plants were air dried, milled and used for extraction. Seeds were used to establish *in vitro* cultures.

### Establishment of shoot culture

Shoot cultures were initiated from seeds in 2013. The seeds were surface sterilized according to the method described in [8, 34] and were germinated on Murashige and Skoog (MS) medium [35] without plant growth regulators (PGRs), solidified with 0.7% agar (w/v). Seedlings were transferred on MS medium supplemented with benzylaminopurine (BAP) 1 mg/L, α-naphthaleneacetic acid (NAA) 0.5 mg/L and gibberellic acid (GA_3_) 0.25 mg/L for multiplication. This PGRs variant was selected on the basis of our previous experience [9, 36]. Little shoots were cultured in 250 ml Erlenmeyer flasks in a growth room at 25±2°C under constant light conditions (continuous lighting, 16 μmol∙m^-2^s^-1^) for a number of 6-week subculture periods. Shoot cultures were used as an experimental material for subsequent biotechnological research.

### Selection of the growth and production medium

*M*. *melissophyllum* shoot cultures were cultivated on ten variants of MS medium (Table 1) (fresh shoots– 3 g/flask; 300 ml Erlenmeyer flasks– 100 ml of the medium solidified with agar). The biomass was harvested in triplicate after 1, 2, 3, 4, 5, 6 weeks, dried at 40°C (the vacuum dryer SPU 250, ZEAMiL Horyzont, Kraków, Poland) and weighed. The growth index was calculated:

GI=(finaldryweight−inoculumdryweight)/inoculumdryweight.

**Table 1:** Variants of MS medium to select the best growth medium.

PGRs [mg/L]	Medium variant									
	1	2	3	4	5	6	7	8	9	10
BAP	1	2	1	1	2	2	2	3	1	2
NAA	–	–	0.5	1.0	0.5	1.0	2.0	1.0	0.5	1.0
GA_3_	–	–	–	–	–	–	–	–	0.25	0.5

The biomass harvested from the best growth media was subjected to the UPLC-MS/MS analysis. The same experiment, but with the use of a liquid medium (without agar), was carried out for the selected best production medium. Its conditions were: agitated shoot cultures on a rotary shaker (Altel), 140 rpm, the temperature and light conditions–the same as previously mentioned. All experiments were prepared in three independent series.

### Supplementation experiment

The agitated shoot cultures for the supplementation experiment were initiated in 2016 by inoculating 3.0 g fresh mass of shoots in Erlenmeyer flasks (300 ml) containing 100 ml liquid MS medium (PGRs–the same as for the best growth and production medium previously selected). The flasks were placed on a rotary shaker (Altel) at 140 rpm, in the same temperature and light conditions as previously. Two weeks after inoculation, 100 ml of the same liquid medium was added. After another week cultures were supplemented with methyl jasmonate (Sigma-Aldrich), ethephon (Sigma-Aldrich) and L-phenylalanine (Merck) according to the scheme (Table 2).

**Table 2:** The scheme of the medium supplementation.

	Variants							
Compound	A	B	C	D	E	F	G	H
L-Phe [g/l]	–	2.4	–	–	2.4	2.4	–	2.4
MeJa [μM]	–	–	50	–	50	–	50	50
Eth [μM]	–	–	–	50	–	50	50	50

The stock solutions of L-Phe, Eth and MeJa had been prepared earlier. L-Phe and Eth were separately dissolved in water, MeJa was dissolved in 96% (v/v) ethanol and all three solutions were filter sterilized (Millex^®^ Millipore, 0.22 μm) before being administered to the medium. Variant A was considered as a control. Variants without MeJa, Eth or L-Phe were supplemented with sterile ethanol or water, respectively. Ethanol in the culture medium achieved a final concentration of 0.1% (v/v). The biomass and the medium (100 ml/flask) were harvested after 2 and 8 days, frozen and lyophilized. The dried biomass and medium were used to prepare extracts for the UPLC-MS/MS analysis. The experiments were performed with three independent samples for biological replicates.

### Extraction

The dried biomass from *in vitro* cultures and dried aerial parts of ground plants were milled (mortar) and 1 g of each sample was extracted with boiling methanol (2 x 100 ml, 2 h). Extracts were combined (200 ml) and concentrated to dryness (rotary vacuum evaporator Büchi, at 40°C). Residues were dissolved in 10 ml of methanol (HPLC grade, Merck). Residues of the lyophilized media were also dissolved in 10 ml of methanol (HPLC grade, Merck). All methanol solution samples were filtered (syringe filters Millipore, Millex-GP, 0.22 μm). All filtered samples were used for the UPLC-MS/MS analysis.

### UPLC-MS/MS analysis of iridoids

The developed UPLC method was chosen specifically for the examined compounds and it guaranteed that well shaped peaks were obtained. No interference that could have an influence on the obtained results was possible because the MRM method was used.

Based on the regression analysis and Mandel’s fitting test (p < 10^−6^), it was assumed that the calibration data fitted the quadratic model well. The correlation coefficient and determination coefficient (r^2^, R^2^) obtained for the model were over 0.99. The y-intercept of the quadratic equation was statistically significant (p < 0.004). The distribution of the residuals can be approximated well with a normal distribution as it is shown by p-value of the normality test (Shapiro-Wilk)–p > 0.63.

The sensitivity of the method was good. The LOD and LOQ values for harpagide were found to be below 0.03 and 0.7 μg/mL, respectively. Good precision and intermediate precision with %RSD less than 9% were observed. The ANOVA test showed no significant differences between analyses conducted on different days (p > 0.48). In all the deliberately varied chromatographic conditions (flow rate, column temperature, mobile phase composition), the examined compounds were adequately resolved, and the order of elution remained unchanged.

The regression analysis results are presented in Table 3.

**Table 3:** Regression analysis results for harpagide.

Statistic	Value
a_0_	0.0691 ± 0.0214 (p < 0.004)
a_1_	0.2848 ± 0.0129 (p < 10^−6^)
a_2_	-0.0104 ± 0,001 (p < 10^−6^)
r	0.996
r^2^	0.992
LOD [μg/mL]	0.023
LOQ [μg/mL]	0.658
Intraday RSD	4.92%
Interday RSD	8.42% (p > 0.48)
Shapiro-Wilk test for residuals	p > 0.63
Mandel’s fitting test	p < 10^−6^

#### Reagents

LC/MS-grade acetonitrile and LC/MS-grade methanol were from Sigma-Aldrich. Formic for LC/MS was from Sigma-Aldrich. HPLC grade water was obtained from HLP 5 (HYDROLAB Poland) apparatus and was filtered through the 0.2 μm filter before the use. Chloramphenicol (≥98%, TLC) was from Sigma. Harpagide analytical standard (≥98%, HPLC) was from Sigma.

#### Preparation of standards

Chloramphenicol internal standard was weighed to 10 mg in a volumetric flask using analytical balance. The volume was brought to 10 mL using methanol to make 1 mg/mL solution. 1 mL of this solution was subsequently diluted in a volumetric flask to 10 mL using methanol to make 100 μg/mL stock. The procedure was repeated to make 10 μg/mL stock solution. Harpagide standard was weighed to 1 mg in a volumetric flask using analytical balance. The volume was brought to 1 mL using methanol to make 1000 μg/mL stock solution. These solutions were stored at -10°C and used to make dilutions for calibration curves.

#### Preparation of calibration samples

A series of dilutions of the 1000 μg/mL harpagide standard solution was prepared by diluting 100 μL of the stock solution with water to make 1 mL, and afterwards diluting 500 μL of the obtained solution again with water to make 1 mL. The procedure was repeated several times to obtain finally a solution with the 0.186 μg/mL concentration of harpagide. 100 μL of the 10 μg/mL chloramphenicol internal standard solution was added to 500 μL of the dilutions of the 1000 μg/mL harpagide standard solution and diluted with water to 1 mL making calibration samples with concentration of chloramphenicol 1 μg/mL and concentration of the harpagide in range 12.5–0.098 μg/mL.

#### Preparation of samples

100 μL or 50 μL of solution was taken from each sample, the 100 μL of 10 μg/mL chloramphenicol internal standard solution was added and brought to 1 mL of water making two dilutions of the stock solutions for each sample. Each dilution was analyzed in triplicate with the UPLC-MS/MS.

#### Quantitative and semi-quantitative UPLC-MS/MS analysis

In order to determine harpagide in the samples, the quantitative UPLC-MS/MS method was developed. Basing on this method the semi-quantitative method for determination of 8-*O*-acetyl-harpagide was created.

The UPLC-MS/MS system consisted of a Waters ACQUITY^®^ UPLC^®^ (Waters Corporation, Milford, MA, USA) coupled to a Waters TQD mass spectrometer (electrospray ionization mode ESI-tandem quadrupole). Chromatographic separations were carried out using the Acquity UPLC BEH (bridged ethyl hybrid) C_18_ column, 2.1 × 100 mm, and 1.7 μm particle size. The column was maintained at 40°C, and eluted under following conditions: linear gradient elution from 100% to 90% of eluent A over 2 min, afterwards linear gradient elution from 90% to 0% of eluent A over 3 min and 100% of eluent B over 1 minute, at a flow rate of 0.3 mL/min. Eluent A: water/formic acid (0.1%, v/v); eluent B: acetonitrile/formic acid (0.1%, v/v). 1 μL of each sample was injected in triplicate.

Waters TQD mass spectrometer was calibrated for the quantitative analysis using harpagide solution in 10 μg/mL concentration at 20 μL/min flow and mixture of eluent A and B 1:1 (v/v) at flow 0.28 mL/min. Optimized settings were as follows: source temperature 150°C, desolvation temperature 350°C, desolvation gas flow rate 600 L/h, capillary potential 3.00 kV, collision gas flow 0.1 mL/min. Cone potential and collision energy were individually optimized for each transition using a harpagide solution or a sample solution containing 8-*O*-acetyl-harpagide (Table 4). Nitrogen was used for both nebulizing and drying gas. Argon was used as collision gas. Traces of analyzed compounds were analyzed using the MRM (Multiple Reaction Monitoring) method.

**Table 4:** Optimized settings for the quantitative analysis of harpagide and 8-*O*-acetyl-harpagide.

Substance	Mode	Rt [min]	Transition	Cone potential [V]	Collision energy [eV]
harpagide	ES-	2.88	363.11 → 201.03^*^	24	14
			363.11 → 183.02	24	20
8-*O*-acetyl-harpagide	ES-	3.51	405.06 → 179.05^*^	30	20
			405.06 → 345.30	30	20
chloramphenicol	ES+	4.20	323.11 → 274.98^*^	12	26
			323.11 → 304.96	12	10

^*^Trace used for quantitation

All analytical data was processed using MassLynx V4.1 software (Waters Corporation, Milford, MA, USA).

#### Method validation

The described method was validated to determine harpagide by the UPLC-MS/MS method according to ICH guidelines.

**Specificity.** To demonstrate the specificity of the developed UPLC method the solution containing the investigated compounds was analyzed.

**System suitability.** Possible interference between different compounds present in the analyzed samples was avoided by using the MRM method. Presence and identity of the compounds were verified by the presence of the peaks with appropriate retention times on both of the analyzed traces.

**Linearity.** The linearity for the investigated compound was assessed by injecting eight separately prepared solutions covering the range of 0.098–12.5 μg/mL of harpagide. During the statistical analysis linear model and linearized nonlinear model (quadratic model) were analyzed:

$$Response=a_{0}+a_{1}c\;\left(linear\;model\right),\;or$$

$$Response=a_{0}+a_{1}c+a_{2}c^{2}\;\left(quadratic\;model\right).$$

In the calculations response was used, defined as Response = AUC·c_int_/AUC_int_, where AUC–area under peak for the analyzed substance, c_int_−concentration of the internal standard in μg/mL, AUC_int_ area under peak for the internal standard. For each compound peak only one trace was used in calculations.

The slope of regression line, y-intercept, standard deviations of the slope and intercept, a correlation coefficient, R^2^ value and standard error of residuals of the calibration curve were calculated using the Statistica v.10 program. Next, to determine whether the residuals have normal distribution, the Shapiro-Wilk statistical test was used. Additionally the Mandel’s fitting test was performed to check the linearity of the calibration curve.

**Limit of detection (LOD) and limit of quantification (LOQ).** Based on the standard error of residuals (SE) and the slope (a) of the calibration plots and following the definition of LOD and LOQ, i.e. LOD–the concentration estimated for the response equal to 10Se and LOQ–the concentration estimated for the response equal to 3.3Se, the LOD and LOQ for examined compounds were estimated.

**Precision.** The repeatability of the method was checked by a sixfold analysis of the 6.25 μg/mL concentration level of harpagide solution. The same protocol was followed for three subsequent different days to study the intermediate precision of the proposed method. The RSD (%) of the peak area of harpagide was calculated. Statistical significance of interday differences was tested with ANOVA.

**Robustness.** To demonstrate the robustness of the method, small changes of flow rate, content of acetonitrile and column temperature were deliberately made in the relation to the optimal values. The mobile phase flow rate was 0.30 mL/min; to study the effect of the flow rate on resolution, the flow rate was changed to 0.27 and 0.33 mL/min. The effect of the column temperature was studied at 36°C and 44°C (instead of 40°C), and the mobile phase composition was changed by +5% from the initial composition.

### Statistical methods

The investigation was prepared according to the rules of the designed experiment [37]. Four controlled factors were selected: a harvesting time (denoted A, due to requirements of Minitab software), a specific concentration of L-Phe (denoted B), MeJa (denoted C) and Eth (denoted D). The factors were controlled at two levels (Table 5).

**Table 5:** Levels of the controlled factors.

Controlled factors		Codes	
Label	Description	-1	+1
A	Harvesting time [days]	2	8
B	L-Phe [g/L]	0	2.4
C	MeJa [μmol]	0	50
D	Eth [μmol]	0	50

Five quantitative outputs were selected: a dry weight (denoted DW, [g]), a concentration of harpagide (denoted Hp, [mg/100 g DW]), a concentration of 8-*O*-acetyl-harpagide (denoted AcHp, [mg/100 g DW]), a total concentration of harpagide and 8-*O*-acetyl-harpagide (denoted Hp&AcHp, [mg/100 g DW]) and a total yield of harpagide and 8-*O*-acetyl-harpagide per flask (denoted FlaskProd, [mg]).

The two-level full factorial 2^4^ experimental design [37] with 16 different treatments was selected as a scheme for the experiment (Table 6). The design was planned as a balanced one with 3 replications in each treatment.

**Table 6:** Two-level full factorial experimental design 2^4^ for 4 factors.

Treatment	A	B	C	D
1	-	-	-	-
2	+	-	-	-
3	-	+	-	-
4	+	+	-	-
5	-	-	+	-
6	+	-	+	-
7	-	+	+	-
8	+	+	+	-
9	-	-	-	+
10	+	-	-	+
11	-	+	-	+
12	+	+	-	+
13	-	-	+	+
14	+	-	+	+
15	-	+	+	+
16	+	+	+	+

The factor settings are presented as coded values in Yates’s manner (-/+) [37]

The fixed effects model was used to analyze the obtained data [37]. Initially, the homogeneity of a variance was tested for each outcome. If heteroscedasticity was detected, the Box-Cox transformation [37] was used and further analysis was carried out for the transformed output.

The initial model was a fixed effects model with interactions up to and including the fourth order. The analysis of effects, combined with the estimation of a pure error on the basis of replications, allowed to determine statistically significant terms of the model [37] visually presented on the Pareto plot. In the next step, insignificant model components were removed and diagnostic tests of the model were used: the normality test of residuals and a lack-of-fit test. The reduced model served as a base for the ANOVA analysis to estimate the individual impact of significant model terms. Tukey-Kramer HSD test [38] was used to identify the homogeneous groups inside treatments and the group related to the maximum of the respective outcome was selected. Finally, the interpretation of the results was developed.

All the computations were performed using Minitab and Statistica software.

## Results

### Selection of the growth and production medium

**Fig 2:**
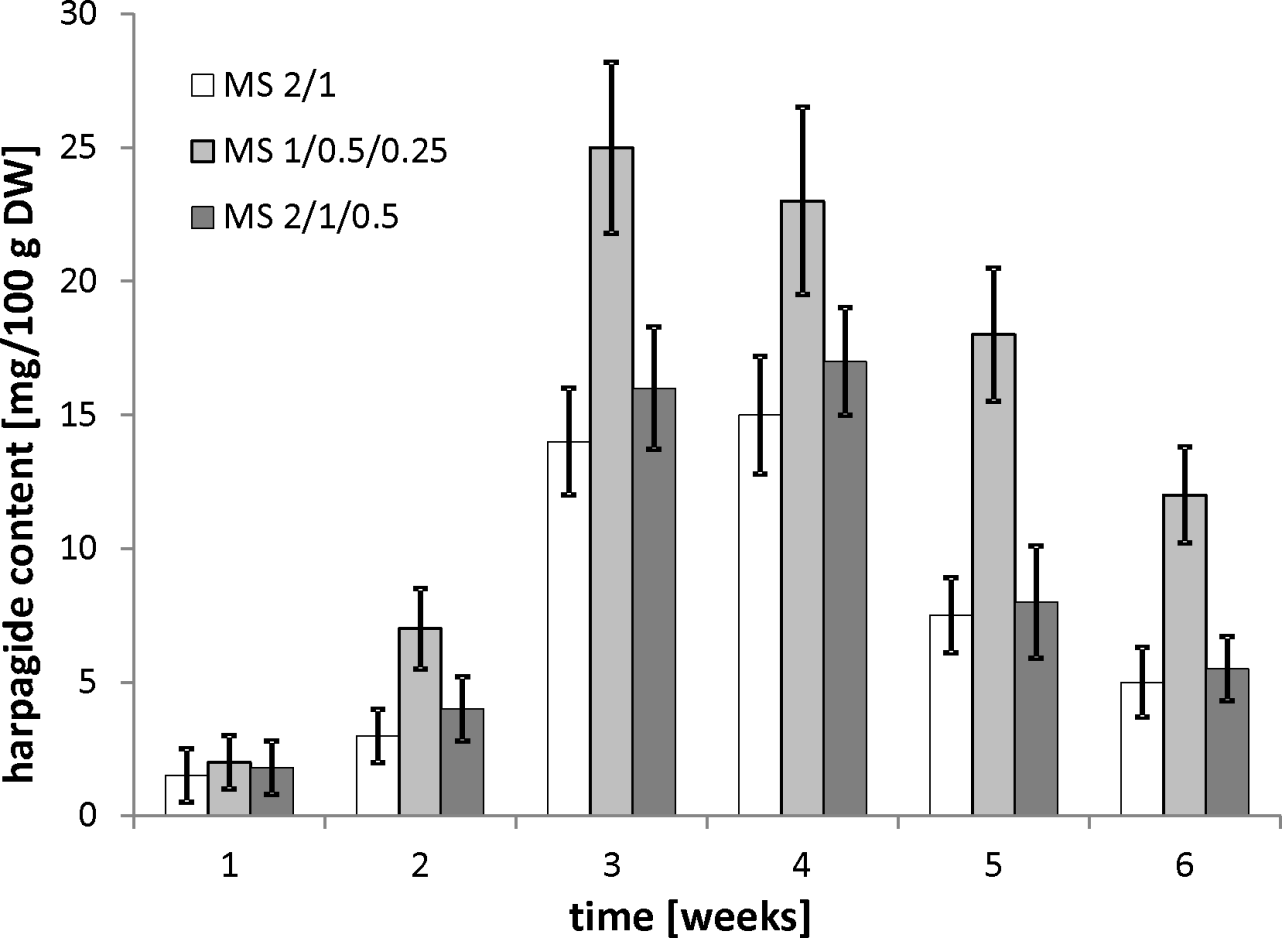
Harpagide content in *M*. *melissophyllum* shoot cultures. Biomass collected after 1, 2, 3, 4, 5 and 6 weeks cultivated on agar MS media. Variants previously selected (Table 7) as the best growth media: MS 2/1 (BAP– 2 mg/L, NAA– 1 mg/L), MS 1/0.5/0.25 (BAP– 1 mg/L, NAA– 0.5 mg/L, GA_3_−0.25 mg/L), MS 2/1/0.5 (BAP– 2 mg/L, NAA– 1 mg/L, GA_3_−0.5 mg/L).

**Fig 3:**
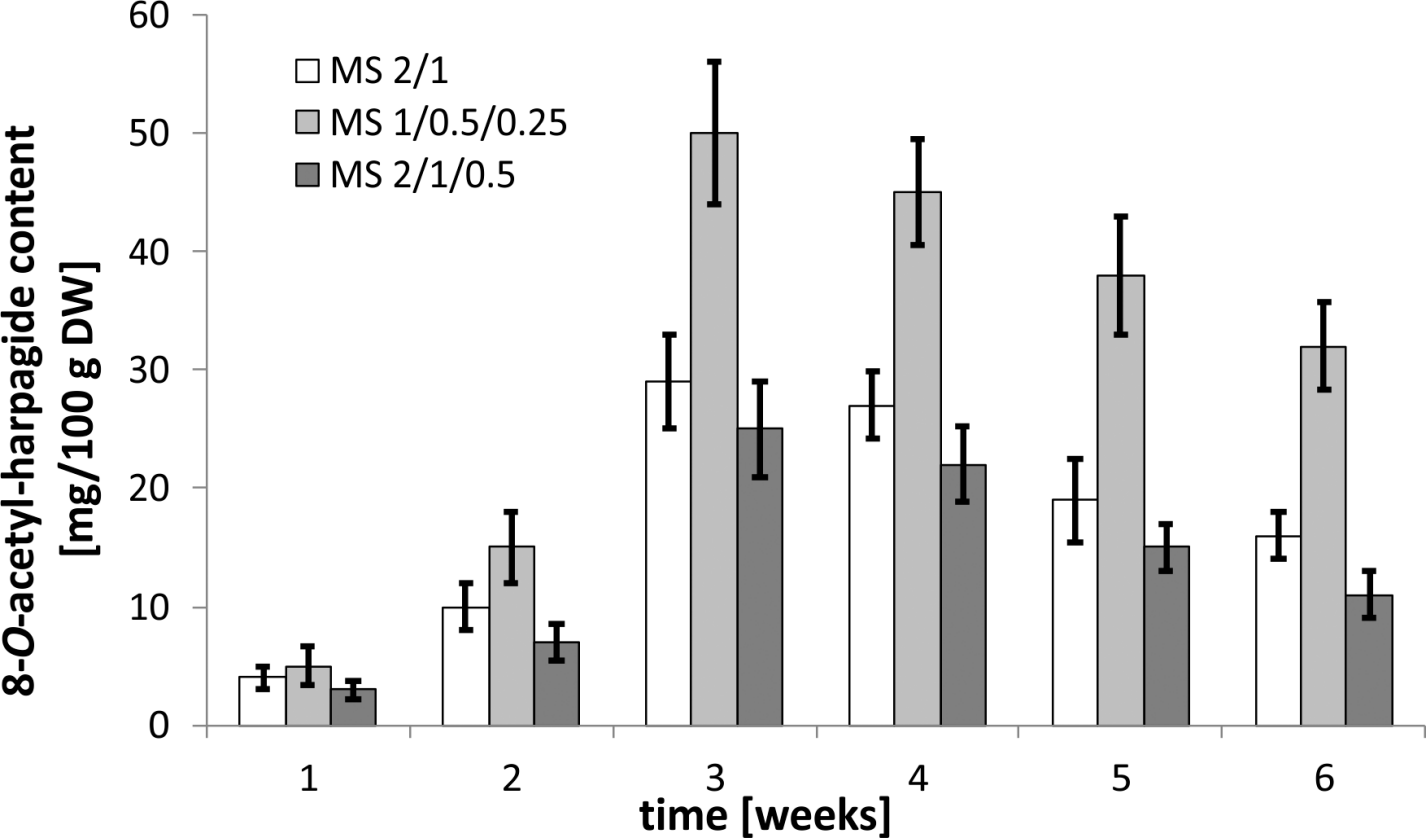
8-*O*-acetyl-harpagide content in *M*. *melissophyllum* shoot cultures. Biomass collected after 1, 2, 3, 4, 5 and 6 weeks cultivated on agar MS media. Variants previously selected (Table 7) as the best growth media: MS 2/1 (BAP– 2 mg/L, NAA– 1 mg/L), MS 1/0.5/0.25 (BAP– 1 mg/L, NAA– 0.5 mg/L, GA_3_−0.25 mg/L), MS 2/1/0.5 (BAP– 2 mg/L, NAA– 1 mg/L, GA_3_−0.5 mg/L).

**Fig 4:**
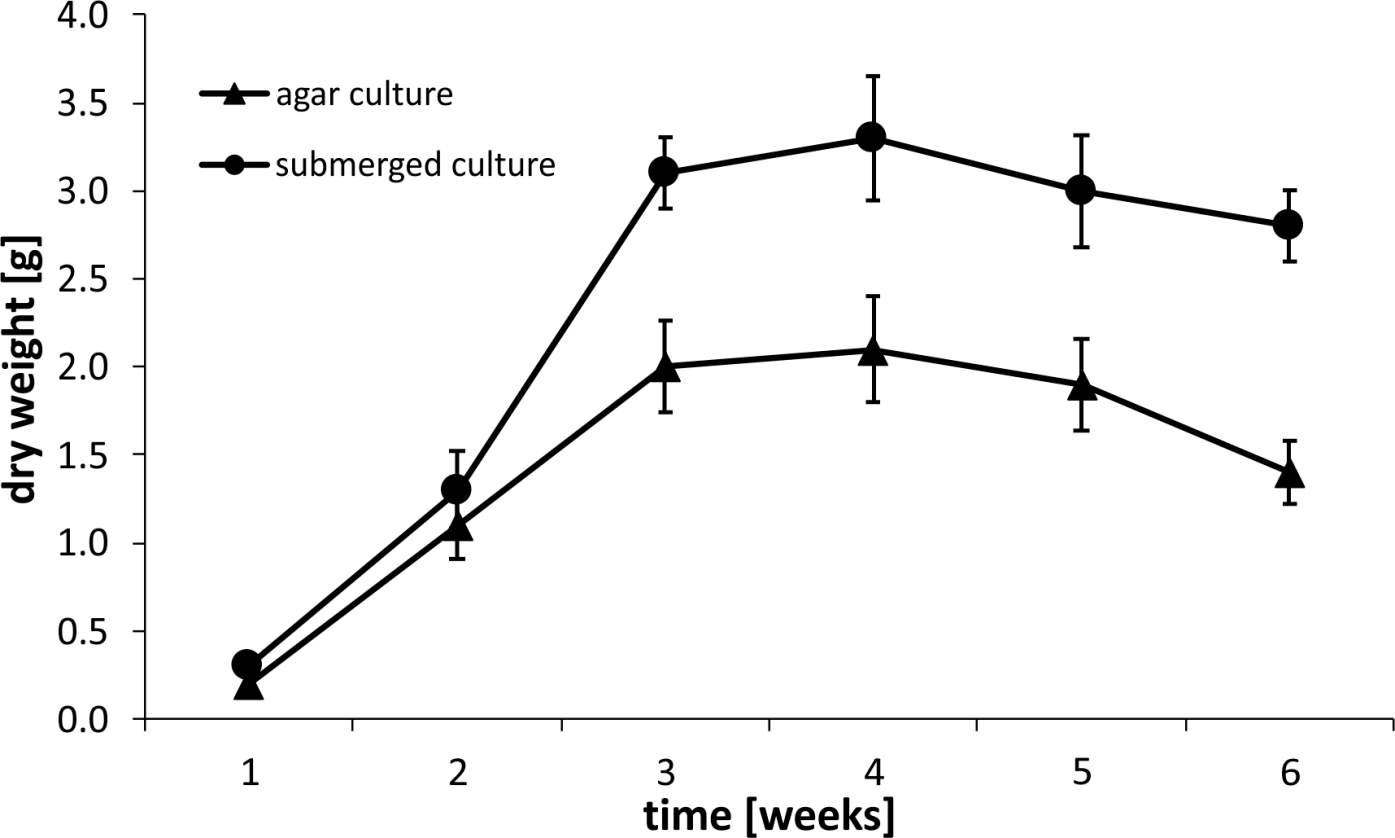
Dry weight of the biomass. Material collected after 1, 2, 3, 4, 5 and 6 weeks from *M*. *melissophyllum* shoot cultures cultivated on agar and liquid MS medium supplemented with BAP– 1 mg/L, NAA– 0.5 mg/L and GA_3_−0.25 mg/L.

**Fig 5:**
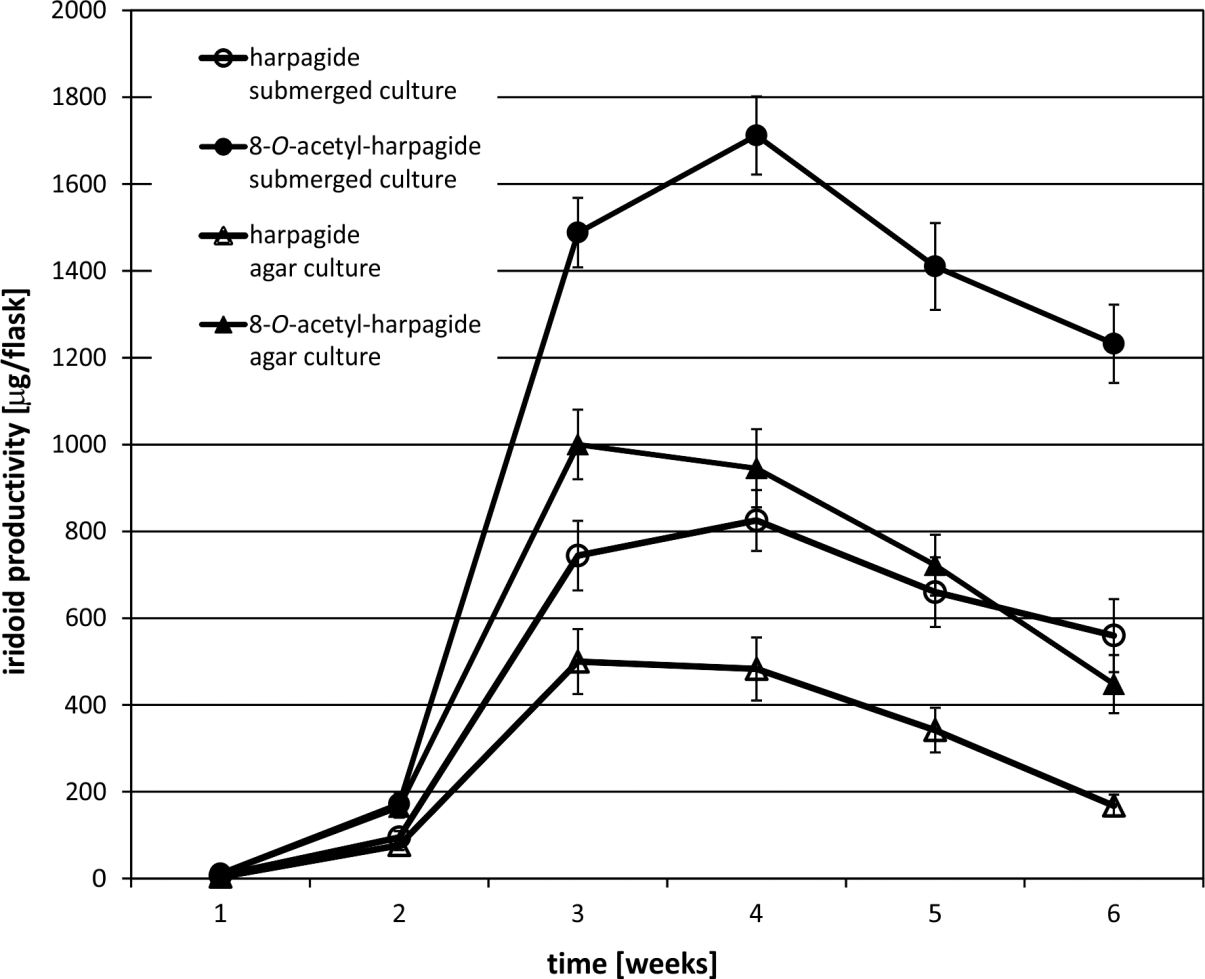
Iridoid productivity in *M*. *melissophyllum* agar and submerged shoot cultures. Biomass collected after 1, 2, 3, 4, 5 and 6 weeks (MS medium supplemented with BAP– 1 mg/L, NAA– 0.5 mg/L, GA_3_−0.25 mg/L).

The experiment began by optimizing the culture conditions for *M*. *melissophyllum* shoot cultures prior to elicitation and precursor supplementation. Ten variants of MS medium supplemented with different PGRs combinations were used (Table 7). Three medium variants with the highest biomass growth index (after 4 weeks) were tested for the Hp and AcHp content (Fig 2 and Fig 3). *M*. *melissophyllum* shoot cultures growing on the MS medium supplemented with BAP– 1 mg/L, NAA– 0.5 mg/L, GA_3_−0.25 mg/L both showed the highest growth index and the highest iridoid content (Table 7, Fig 2 and Fig 3). Agitated shoot cultures showed higher biomass growth and iridoid productivity than agar cultures (Fig 4 and Fig 5).

**Table 7:** Ten variants of MS medium used in the experiment to select the best growth and production media for *Melittis melissophyllum* shoot cultures.

PGRs [mg/L]			Growth index^a^			
BAP	NAA	GA_3_	2 weeks^b^	3 weeks^b^	4 weeks^b^	5 weeks^b^
1	–	–	1.2±0.1	1.7±0.2	1.8±0.2	1.6±0.2
2	–	–	1.2±0.1	1.9±0.2	2.0±0.2	1.8±0.2
1	0.5	–	1.3±0.1	2.6±0.2	2.7±0.2	2.5±0.2
1	1.0	–	1.3±0.1	2.4±0.2	2.5±0.3	2.2±0.2
2	0.5	–	1.4±0.2	2.8±0.3	2.9±0.3	2.6±0.2
2	1.0	–	1.6±0.2	3.7±0.3	3.9±0.3	3.4±0.4
2	2.0	–	1.2±0.1	3.0±0.3	3.1±0.3	2.8±0.2
3	1.0	–	1.3±0.1	2.9±0.3	2.9±0.3	2.4±0.2
1	0.5	0.25	1.8±0.2	4.0±0.4	4.2±0.4	3.8±0.3
2	1.0	0.50	1.4±0.2	3.5±0.4	3.7±0.3	3.2±0.3

All experiments were prepared in three independent series. Data are mean ± SD.

^a^Growth index GI = (final dry weight–inoculum dry weight) / inoculum dry weight

^b^Growth index was calculated for biomass harvested after 2, 3, 4 and 5 weeks.

### Supplementation experiment

The MS medium supplemented with BAP– 1 mg/L, NAA– 0.5 mg/L and GA_3_−0.25 mg/L was used in the supplementation experiment. The study investigated the influence of the addition of methyl jasmonate (MeJa, 50 μM), ethephon (Eth, 50 μM) and L-phenylalanine (L-Phe, 2.4 g/L of medium), in different combinations (one-, two- and three-component variants, B-H), on the content of harpagide and 8-*O*-acetyl-harpagide in *M*. *melissophyllum* shoot cultures (see Table 2). Variant A was a control culture without any supplementation.

#### The influence of MeJa, Eth and L-Phe on the biomass weight

**Fig 6:**
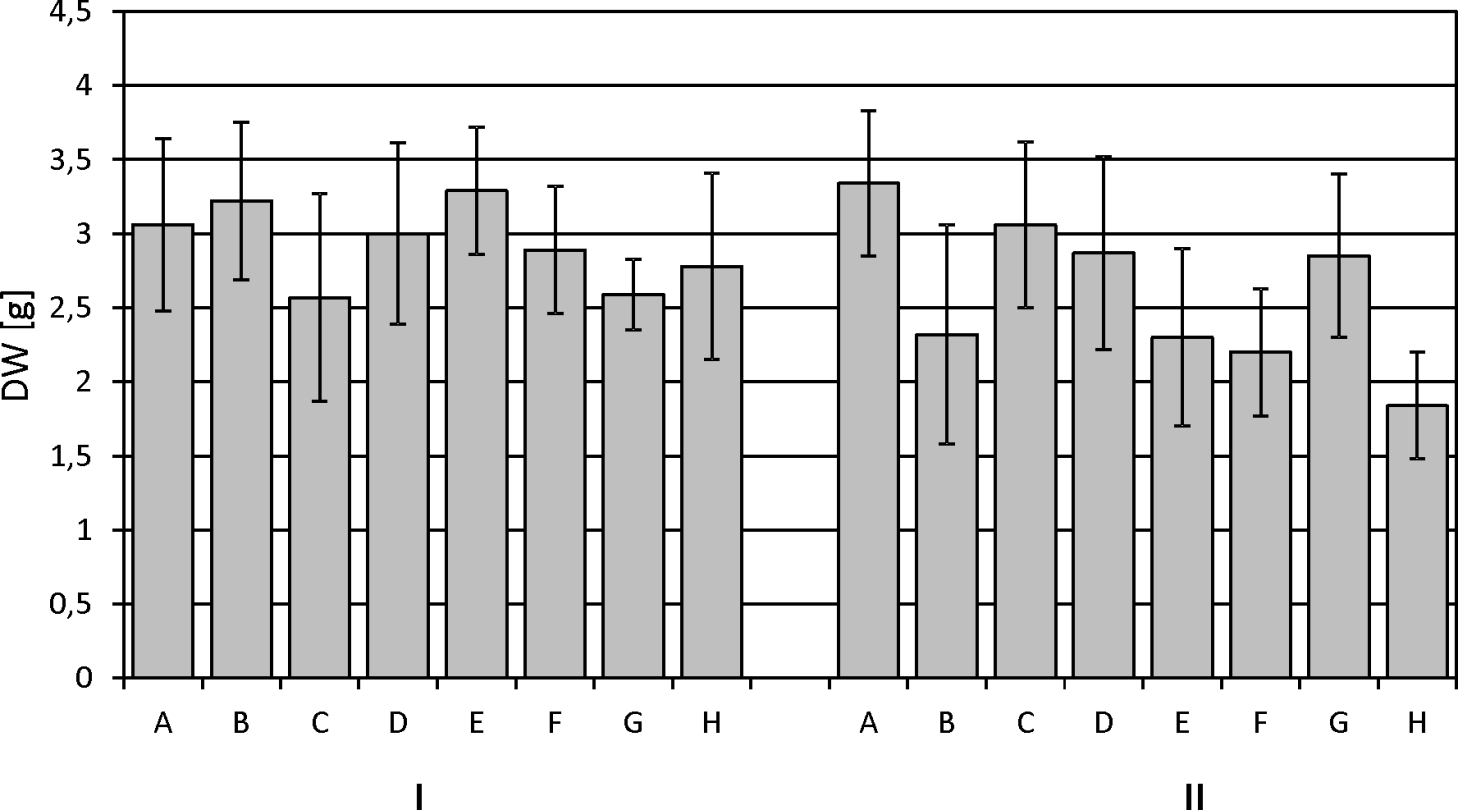
Dry biomass collected from *Melittis melissophyllum* agitated shoot cultures. Different variants of supplementation A-H (see Table 2); harvesting time: I– 2^nd^ day and II– 8^th^ day after supplementation.

Comparing the average dry weight of the biomass harvested 2 days after the modification of the medium, it can be stated that it was higher, to a small extent, for variants B and E, and for variants C, F, G, H a little lower than the average weight of the biomass harvested from the control culture (variant A) (Fig 6).

The differences, however, were not statistically significant. For variant D, the corresponding values were similar to variant A. The average dry weight of the plant material harvested after 8 days showed greater variability. It was the highest for variant A representing the control. The addition of L-Phe, MeJa and Eth (in various combinations) caused a slowdown in the growth of the cultures. A particularly strong reduction in weight could be observed for the variants containing L-Phe in their composition (variants B, E, F, H). The lowest average weight of the biomass was found for variant H (L-Phe together with MeJa and Et), which was about 40% lower than the average weight of the biomass harvested from the control culture (variant A). Moreover, for variants E, F, G and H, slight partial browning of the plant material was observed. This may indicate some damage to plant tissues and consequently lead to the release of iridoids into the medium. The HPLC analysis of the media collected from the cultures confirmed this supposition. The presence of the metabolites in the medium was found for those particular variants (E, F, G, H) harvested eight days after the time of modification.

#### The influence of MeJa, Eth and L-Phe on the harpagide and 8-*O*-acetyl-harpagide accumulation

**Fig 7:**
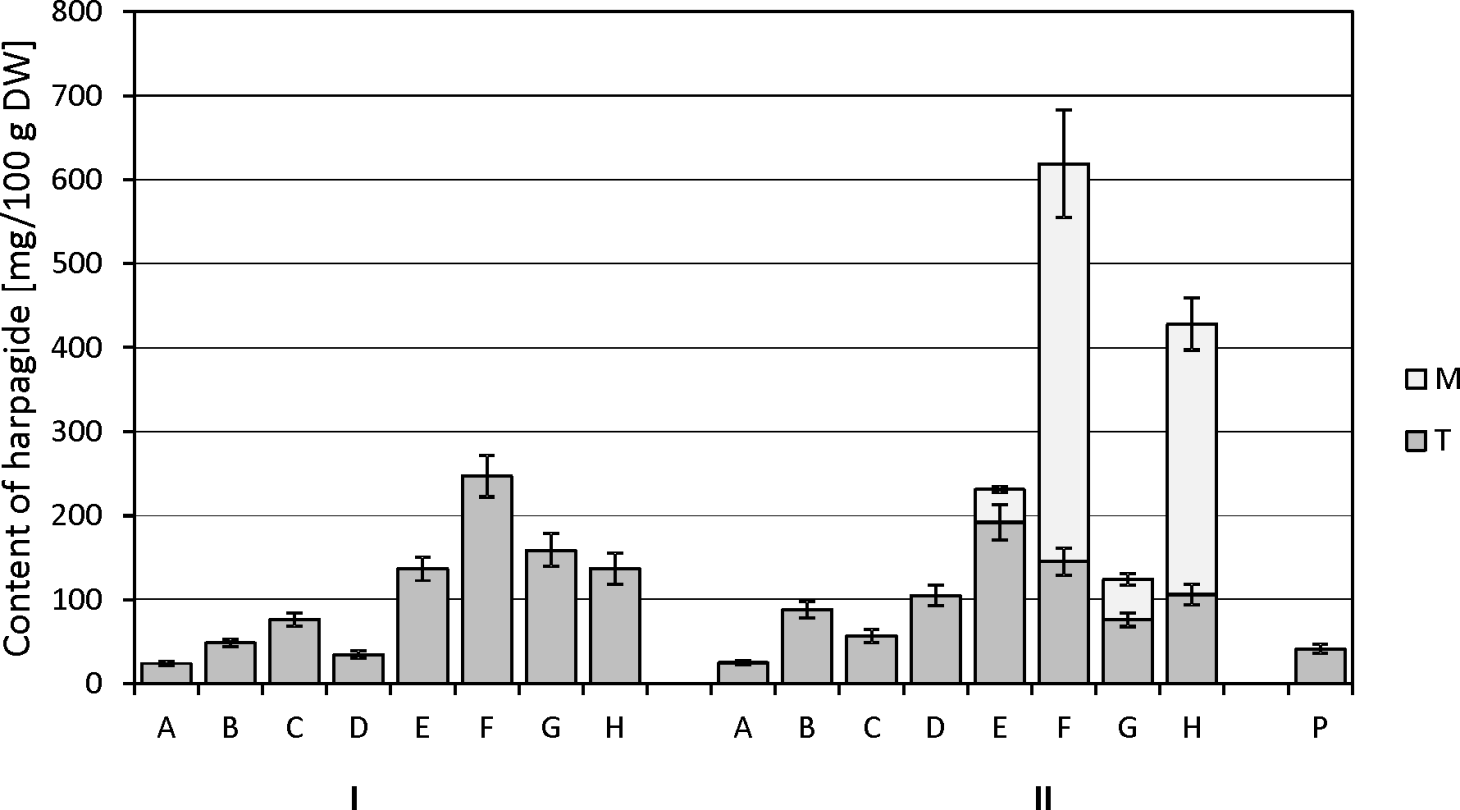
Harpagide content in *Melittis melissophyllum* agitated shoot cultures and ground plant. Shoot cultures with different variants of supplementation A-H (see Table 2) vs. a ground plant (P). Other symbols: M–medium; T–tissue = biomass of shoots; harvesting time: I– 2^nd^ day and II– 8^th^ day after supplementation.

It was found that each modification of the medium, consisting in the addition of L-Phe, MeJa and Eth in various one-, two- and three-component combinations (variants B-H), increased the accumulation of harpagide (Hp) in the biomass, in comparison with the control culture (variant A) (Fig 7).

In the plant material harvested two days after the modification of the medium, the highest Hp content was observed for variant F (L-Phe and Eth), which was 247.3 mg/100 g DW, and was approx. 10 times higher than in the control. The addition of L-Phe in conjunction with MeJa (variant E) or L-Phe in conjunction with both elicitors (variant H) was less advantageous than variant F, and caused similar increases in accumulation– 136.7 and 137.0 mg/100 g DW respectively (5.7-fold increase). Individual additions of only L-Phe (variant B) or only Eth (variant D) resulted in only a slight increase in accumulation– 2-fold and 1.4-fold, respectively.

Comparing the average Hp content in the biomass harvested 8 days and 2 days after the modification of the medium, it can be stated that only for variants B, D and E there was an increase in the accumulation of the metabolite, and for the other variants–a decrease in content. For variants E, F, G and H, one must, however, take into consideration the fact that a portion of Hp had been released into the medium. For variants E and G, the greater part of Hp was located in the biomass than in the medium, whereas for variants F and H the amount of the compound was approx. 3 times greater in the medium than in the plant material. The highest average Hp content in the biomass was found for variant E (192 mg/100 g DW). Considering the total amount of the compound (in both plant material and medium), the highest accumulation of it (far exceeding the content in the biomass harvested 2 days after modification) was observed for variants F and H– 619 and 428 mg/100 g DW respectively (24.7-fold and 17.1-fold increase in content respectively, as compared with the control culture).

**Fig 8:**
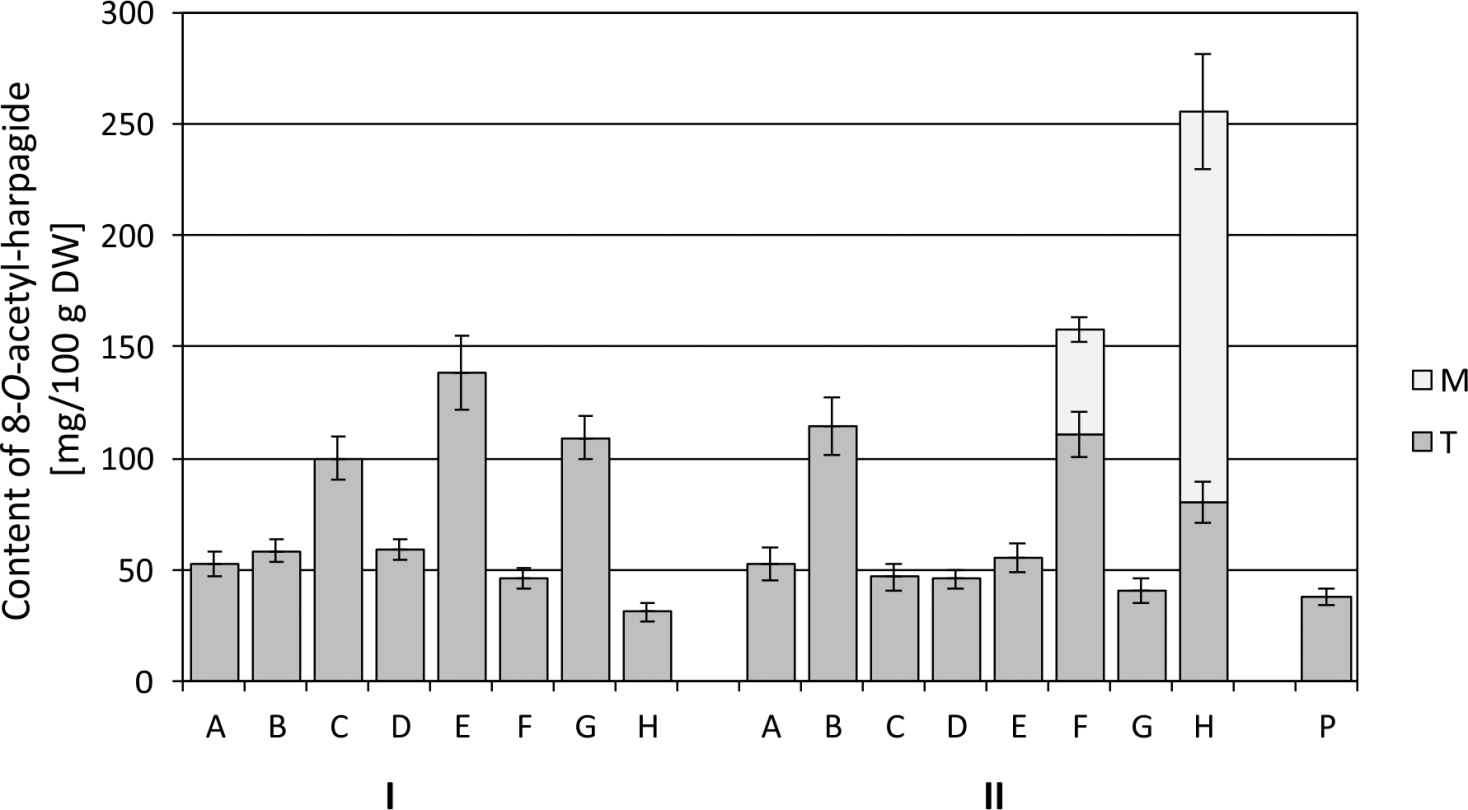
8-*O*-acetyl-harpagide content in *Melittis melissophyllum* agitated shoot cultures and ground plant. Shoot cultures with different variants of supplementation A-H (see Table 2) vs. a ground plant (P). Other symbols: M–medium; T–tissue = biomass of shoots; harvesting time: I– 2^nd^ day and II– 8^th^ day after supplementation.

The average 8-*O*-acetyl-harpagide (AcHp) content in the control cultures (variant A) was approx. twice as high as the average Hp content in analogous cultures (Fig 7 and Fig 8).

Analyzing the biomass harvested 2 days after modifying the media with the addition of L-Phe, MeJa and Eth, it can be stated that a marked increase in the accumulation of AcHp occurred only for variants C, G and E (1.9-fold, 2.1-fold and 2.6-fold respectively), reaching the maximum average content of 138 mg/100 g DW for variant E. After another 6 days (i.e. 8 days after the modification of the media), the situation changed, and an increase in AcHp in the biomass was found for other variants–B, F and H, where it reached the maximum of 114.2 and 110.7 mg/100 g DW (variants B and F respectively). However, considering the total AcHp content (in both the biomass and medium), the highest average content of 255.4 mg/100 g DW was found for variant H.

**Fig 9:**
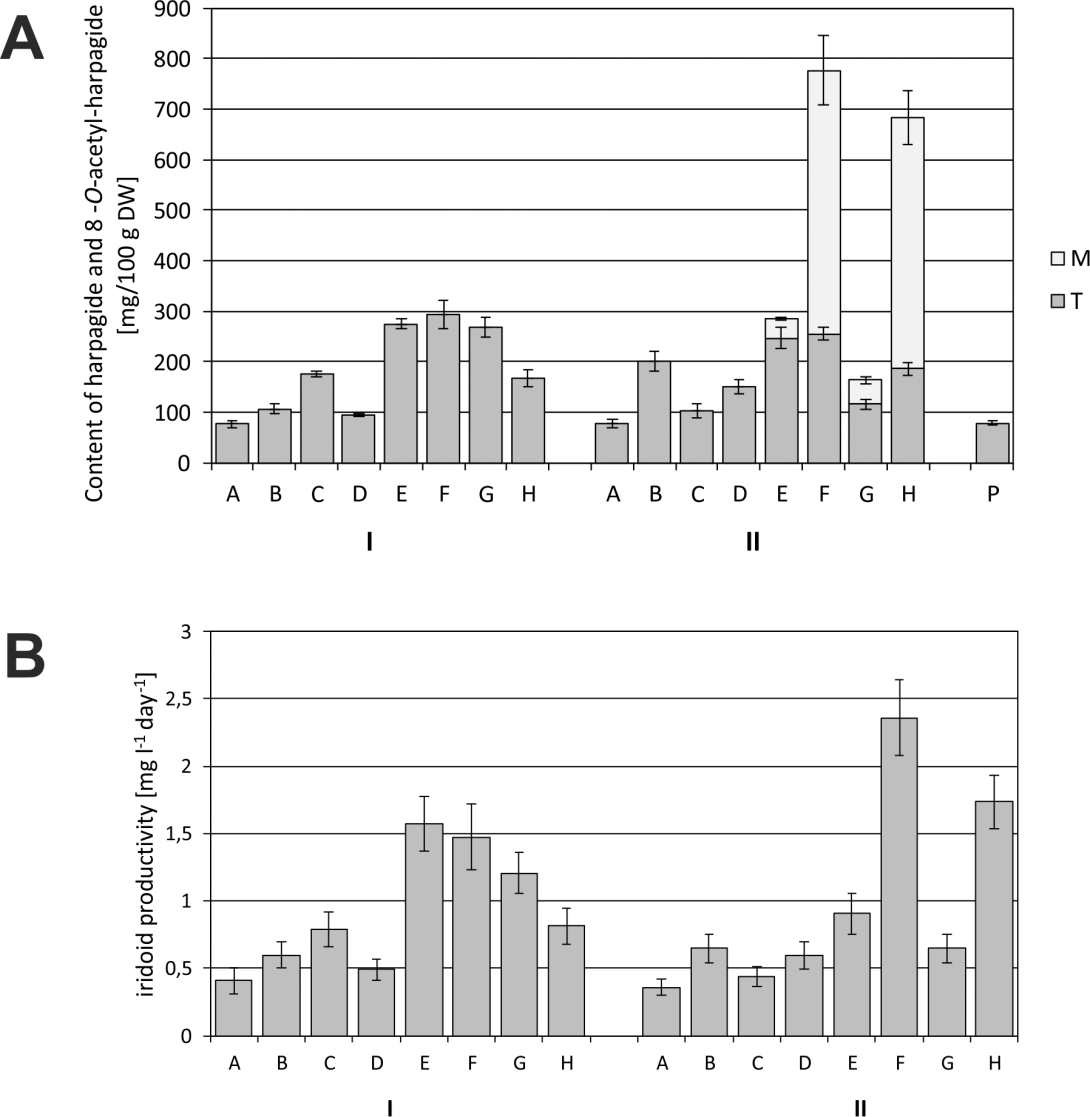
**Total content of iridoids in *Melittis melissophyllum* agitated shoot cultures and ground plant (a), and iridoids productivity (b).** Shoot cultures with different variants of supplementation A-H (see Table 2) vs. a ground plant (P). Other symbols: M–medium; T–tissue = biomass of shoots; harvesting time: I– 2^nd^ day and II– 8^th^ day after supplementation. Productivity expressed as mg of harpagide and 8-*O*-acetyl-harpagide (sum of iridoids) per liter of a medium per day of the culture.

AcHp is an ester derivative of Hp. They share the same biosynthetic pathway, and therefore the accumulation of the two compounds in the cultivated cultures should be considered jointly (Fig 9).

Analyzing the combined amounts of Hp and AcHp in the plant material harvested 2 days after media modifications, the highest increase in accumulation (from 3.5 to 3.8-fold) was found for variants G, E and F, where it reached the maximum of 293.3 mg/100 g DW (Fig 9A). The highest productivity was found for variants E and F (1.572 and 1.474 mg l^-1^ day^-1^, respectively), 3.9 and 3.6-fold higher, respectively, comparing with the control culture–variant A (Fig 9B).

After another 6 days, the highest combined amount of the two compounds (in the biomass and medium) was found for variants F and H– 776.7 and 683.4 mg/100 g DW respectively. In comparison with the control culture there was, respectively, a 10-fold and 8.8-fold increase in the combined amount of the two metabolites (Fig 9A). The highest productivity was found for variants F and H (2.357 and 1.734 mg l^-1^ day^-1^, respectively), 6.3 and 4.9-fold higher, respectively, comparing with the control culture–variant A (Fig 9B).

## Discussion

### In vitro cultures versus soil-grown plants

The content of iridoids in aerial parts of *M*. *melissophyllum* from both Botanical Gardens did not differ significantly in statistical terms. The Hp content in the shoots of the flowerless (not flowering) soil-grown plant of *M*. *melissophyllum* was 41.3 mg/100 g DW, and the AcHp content– 37.9 mg/100 g DW. These amounts were, respectively, approx. 72% higher and approx. 27.8% lower than in the biomass harvested from the control culture (variant A). This is quite different in the case of *Rehmannia elata* N.E. Brown ex Prain, a plant that also contains Hp. Shoot cultures of this species accumulate from 13 to 25 times more Hp than the shoots of the soil-grown plant [39]. The stems of soil-grown *M*. *melissophyllum* contain only small amounts of Hp and AcHp, 2.7 mg/100 g DW and 2.1 mg/100 g DW respectively, many times lower than the shoots (about 15 and about 18 times less, respectively). A similar relationship was observed for soil-grown plants of *Scrophularia nodosa* L. [40]. The leaves of this species contain approx. 13 times more harpagoside (Hpd, a derivative of harpagide) than the stems. The species *Scrophularia yoshimurae* Yamazaki accumulates Hpd in both the roots and the aboveground parts [41]. Changes in the amounts of this metabolite were observed depending on whether the plant was flowering or had no flowers. For many secondary metabolites belonging to different chemical groups, seasonal changes in the accumulation of them were observed, depending on the growth stage of the plant (e.g. [23, 24]).

The medium variants that are optimal for the accumulation of the tested metabolites, in the case of harvesting the biomass 8 days after the modification of the medium, are variants F and H. The amounts of Hp and AcHp in the biomass from in vitro cultures are then many times higher than in the leaves of the soil-grown plant: about 15 times and about 4 times respectively for variant F, and about 10 times and almost 7 times respectively for variant H.

### The influence of MeJa, Eth and L-Phe on harpagide and harpagide derivatives accumulation

Taking into consideration the composition of variants F and H, it can be concluded that the crucial addition is that of L-Phe together with Eth (variant F). The use of L-Phe with two elicitors (variant H) more considerably increases the accumulation of AcHp than Hp, whereas the use of L-Phe only in conjunction with MeJa (variant E) is not optimal for either of the two metabolites.

The use of elicitors is a known method of enhancing in cultures *in vitro* the accumulation of metabolites [42] from various chemical groups, including terpenoide compounds, e.g. [43–46]. It is very important to select the right concentration of the elicitor and the time of biomass harvesting. Daidzin production in hairy root cultures of *Psoralea carylifolia* increased 2.8-fold with 1 μM jasmonic acid (JA) treatment after second week and 7.3-fold with 10 μM JA elicitation after 10^th^ week compared to untreated control [47]. JA was also an effective elicitor for adventitious root cultures of *Andrographis paniculata*. Andrographolide production after the first week increased 3.5-fold with 1 μM JA and 10.8-fold with 25 μM JA compared to control [46]. The effect of the elicitor on the accumulation of secondary metabolites also depends on the type of in vitro culture. Andrographolide production in multiple shoot cultures of *Andrographis paniculate* increased 2.6-fold with 1 μM JA after fifth week but JA treatment at 25 μM promoted only 3.3-fold enhancement in metabolite content after eighth week compared to control [45].

Sometimes, a better result is obtained through the simultaneous use of two elicitors. According to Bae, Choi [48], the combined application of 50 μM Eth and 100 μM MeJa has the most beneficial effect on increasing the accumulation of ginsenosides in adventitious root cultures of *Panax ginseng* C.A. Meyer.

It is puzzling that the addition of L-Phe increases the accumulation of Hp and AcHp to a particularly high extent when it is added together with Eth, or Eth and MeJa. L-Phe is an aromatic amino acid biosynthesized via the shikimic acid pathway. It is a precursor of many groups of chemical compounds, in particular derivatives of phenylpropane. The metabolic pathway leading to the biosynthesis of harpagide is still not fully understood [49–52], but there is no indication that it is in some way connected with the shikimic acid pathway. Therefore, in our experiment, we can exclude the effect of L-Phe as that of a precursor. The amino acid is added in a relatively large amount– 2.4 g/l of medium. Perhaps it causes, like sugars, osmotic stress that can exert a stimulating action on the biosynthesis of metabolites. Another hypothesis might suggest that the presence of the amino acid may enhance the synthesis of some enzymes involved in the iridoids biogenetic pathway.

### The shoot cultures of *M*. *melissophyllum* versus in vitro cultures of other species

There are not many publications concerning research on increasing the accumulation of Hp in cultures *in vitro*. One of such studies describes the influence of growth regulators on the accumulation of several metabolites (incl. Hp) in shoot cultures of *Rehmannia elata* N.E. Brown ex Prain [39]. The medium used was MS [35] containing indoleacetic acid (IAA; 0.57 μM) and cytokinin–BAP, kinetin or 2-isopentenyladenine (2iP) at various concentrations. The highest Hp content of 1.108 mg/g DW was found for the variant supplemented with 2iP at 4 μM. The amount of the compound was twice as high as in the control culture without the growth regulators added. Comparing the results obtained for *M*. *melissophyllum* shoot cultures with the results presented above, it can be noticed that the Hp content in the biomass harvested from the optimal variant (F) is approx. 6 times higher than that for *R*. *elata* cultures.

In another study [53], the investigation concerned the influence of the elicitors, MeJa and salicylic acid, on the accumulation of metabolites (incl. Hp) in hairy root cultures of *Rehmannia glutinosa* Libosch. The highest Hp content of 0.136 mg/g DW was obtained 72 hours after the addition of 150 μM MeJa. This content was 7.5 times higher than the one for the control culture, without the addition of elicitors. The simultaneous addition of MeJa and SA did not produce such a beneficial effect. The maximum Hp content in *M*. *melissophyllum* shoot cultures is up to 45.5 times higher than in the hairy root cultures of *R*. *glutinosa*. The authors [53] found that both MeJa and SA had an inhibitory effect on the growth of hairy root cultures. As a result, the dry weight of the biomass from cultures containing the elicitors is reduced, as compared to control cultures. Similar observations were made for the use of MeJa in shoot cultures of *Exacum affine* [9].

The main compound of the iridoid fraction in the tubers of soil-grown *Harpagophytum procumbens* plants is Hpd. Apart from this compound, other iridoid glycosides, incl. Hp, are present in smaller amounts. According to the European Pharmacopoeia [1], the raw material should contain not less than 1.2% Hpd. For hairy root cultures of *H*. *procumbens*, a study examined the influence of the method of cultivating the culture (in shake flasks and in a 3-litre bubble column bioreactor) on the accumulation of Hpd and Hp in the biomass [54]. In contrast to soil-grown plants, the hairy root cultures were found to produce much more Hp than Hpd: about 7 times more in the flasks, and as much as about 130 times more in the bioreactor. These results generate the idea of trying to cultivate shoot cultures of *M*. *melissophyllum* in a bioreactor in the future. The maximum Hp content in the hairy roots of *H*. *procumbens* was only approx. 4.5 μg/g DW. In other experiments with hairy root cultures of this species [8, 9], the amounts of iridoids in the biomass from the *in vitro* cultures were also considerably lower than in the raw material from the soil-grown plant, and were as follows: 0.32 mg/g DW (Hpd [55]) and 0.66 mg/g DW (total iridoid content [56]). Those results, and also our own results on the shoot cultures of *M*. *melissophyllum*, suggest that it is essential to add elicitors to *in vitro* cultures in order to obtain substantial accumulation of iridoids. In another experiment with the *H*. *procumbens* species [57], the total iridoid content was determined in the tubers harvested from their natural state, in the tubers of plants propagated in *in vitro* cultures, acclimatized and grown in a greenhouse, and in shoots and callus tissue from *in vitro* cultures. In the callus tissue of *H*. *procumbens* [57] there were significant quantities of iridoids, and in the callus culture of *Scrophularia nodosa* L. they were not found to be present at all [40]. The authors [3, 40] suggest that in the undifferentiated cells of the callus the genes responsible for the biosynthesis of iridoids may be inactive. Therefore, it is more advisable to use cultures of plant organs to obtain high accumulation of these compounds.

### Statistical analysis and results

Statistical analysis confirms previously mentioned observations.

#### Dry weight

**Fig 10:**
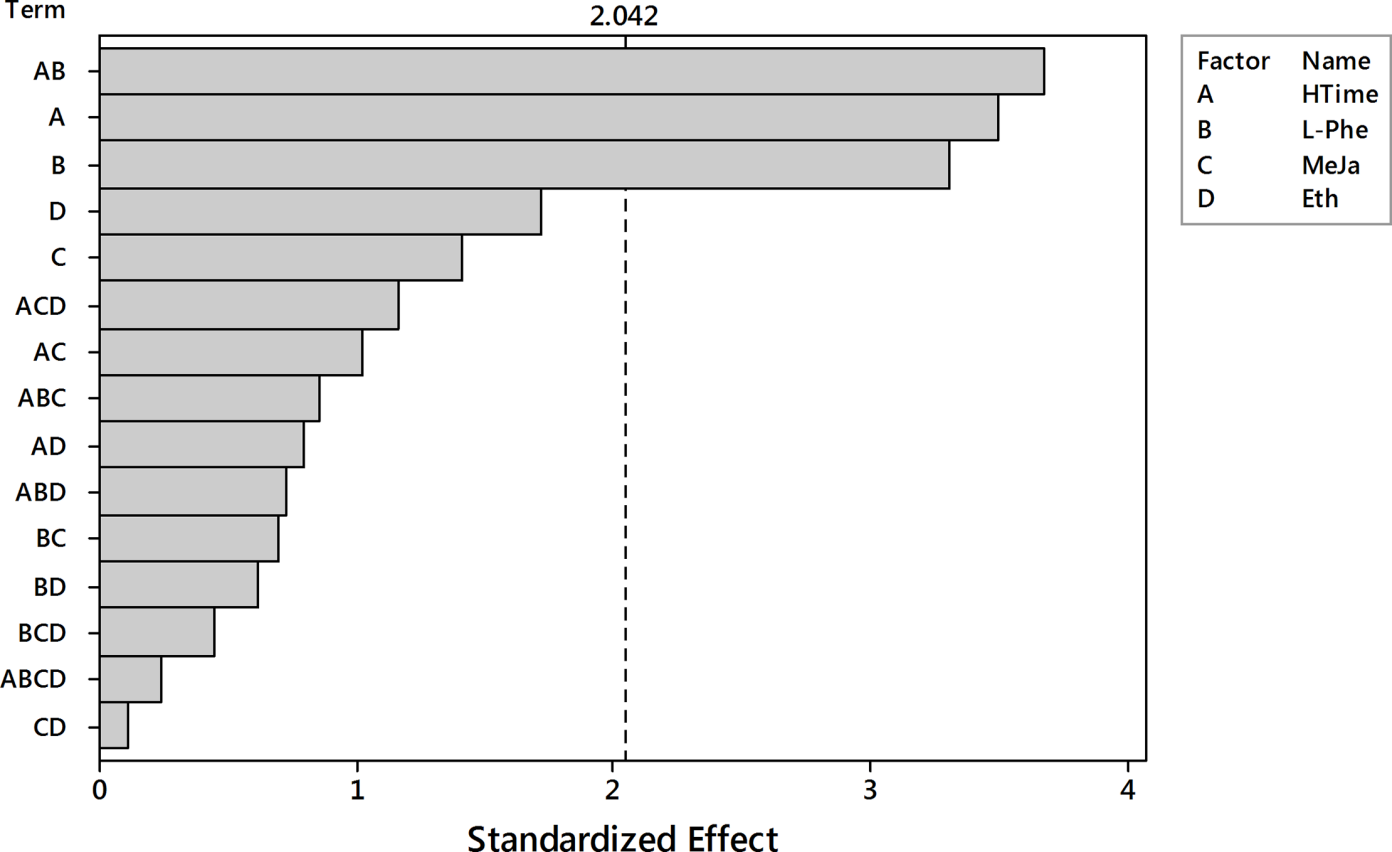
Pareto plot of standardized effects for the dry weight outcome. Four factors (HTime, L-Phe, MeJa, Eth) and their interaction up to the 4^th^ order were considered. Significance level α = 0.05, t = 2.042.

The analysis of the results (48 records) started from the fixed-effects model with interactions up to fourth order. Pareto plot of standardized effects (Fig 10) revealed that only two factors, the harvesting time (HTime) and the L-Phe concentration, and their interaction have the significant impact on the dry weight outcome.

**Fig 11:**
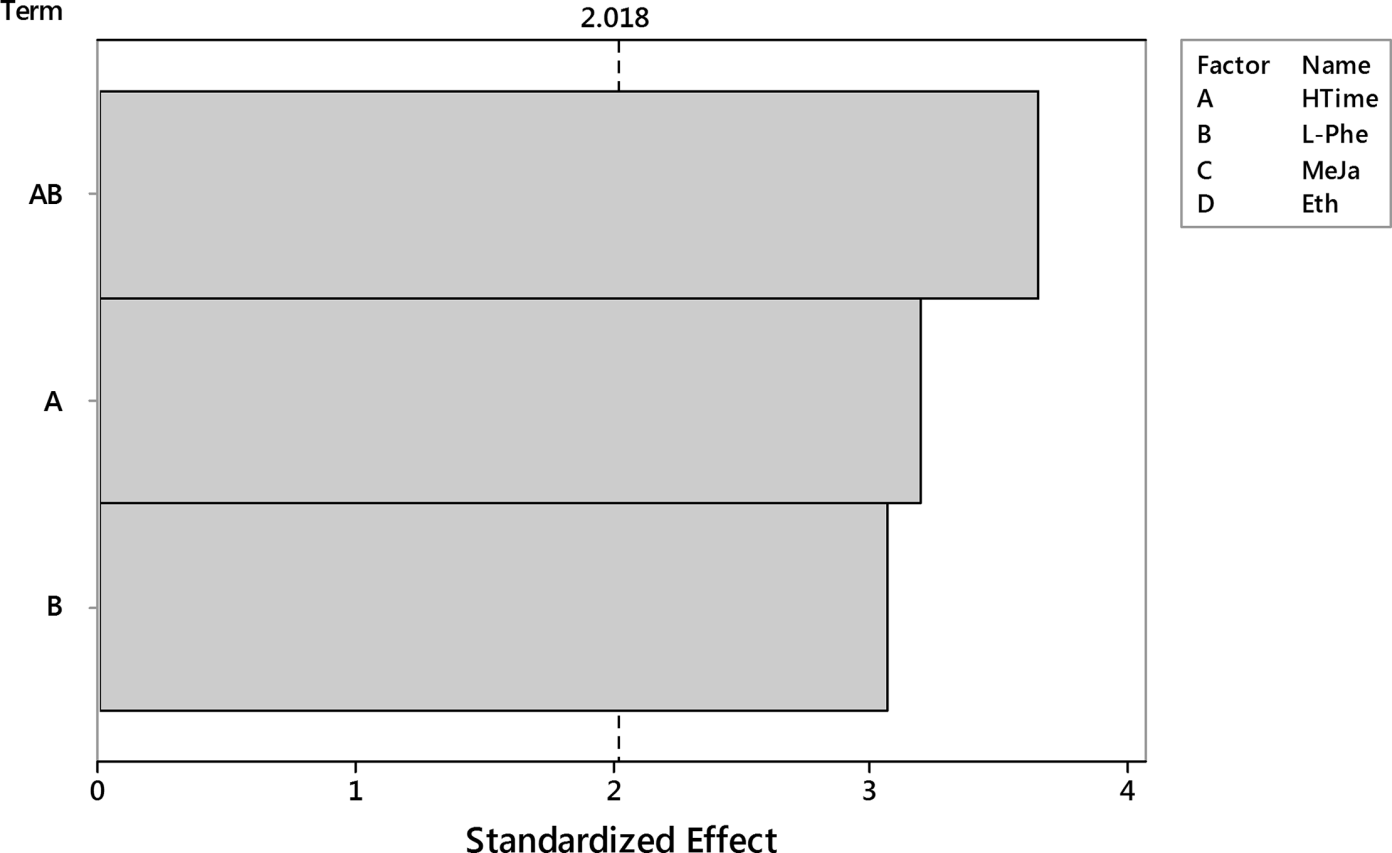
The Pareto plot of standardized effects for the reduced model of DW outcome. Two significant factors (HTime, L-Phe) and their interaction were considered. Significance level α = 0.05, t = 2.018.

The insignificant model terms were removed and the analysis was performed again for HTime, L-Phe and their interaction. Pareto plot of standardized effects (Fig 11) confirmed the statistical significance for all model terms.

ANOVA analysis (Table 8) reported a well fitted model–the lack-of-fit component is not significant (p = 0.681), however the coefficient of determination is rather low R^2^ = 0.43. Such value of R^2^ suggests that 57% of unexplained variance is caused by other factors: controlled, but ignored (because of their insignificance) MeJa and Eth, and also other environmental and material factors not considered here.

**Table 8:** ANOVA analysis for the 2^nd^ order model for two factors and dry weight outcome.

Source	df	SS	MS	F	p
HTime	1	2.572	2.572	10.22	0.003
L-Phe	1	2.372	2.372	9.43	0.004
HTime x L-Phe	1	3.357	3.357	13.34	0.001
Error	42	10.569	0.252		
Lack-of-fit	12	2.472	0.206	0.76	0.681
Pure error	30	8.097	0.270		
Total	45	18.665			

Abbreviations: df–degree of freedom, SS–sum of squares, MS–mean square, F–Fisher’s statistic, p–critical significance level.

**Fig 12:**
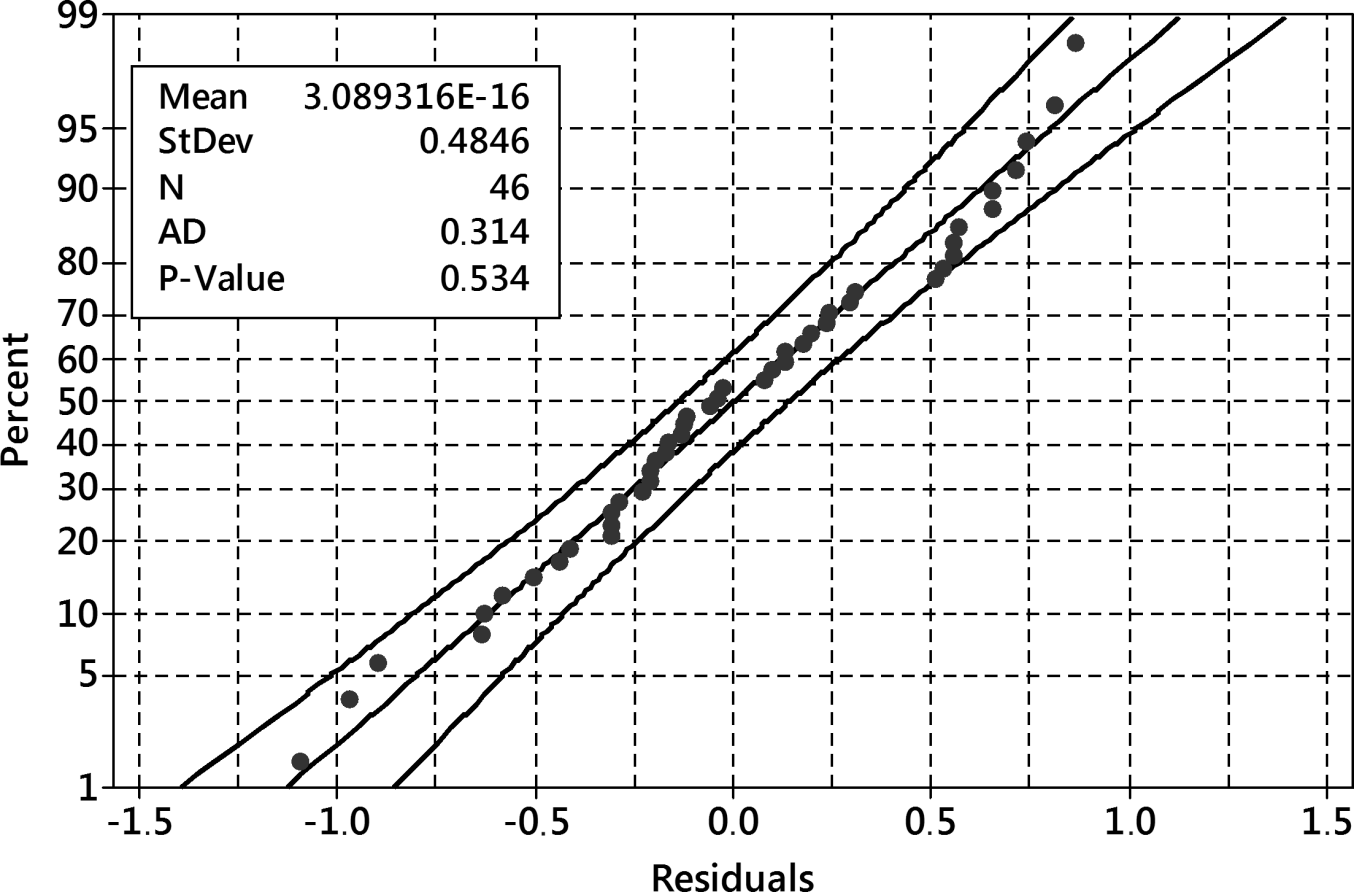
The probability plot of residuals for the reduced model of dry weight outcome. 95% confidence band 95%, Anderson-Darling test of normality p = 0.534.

The model residuals (Fig 12) followed the normal distribution–they passed Anderson-Darling normality test with p = 0.534.

**Fig 13:**
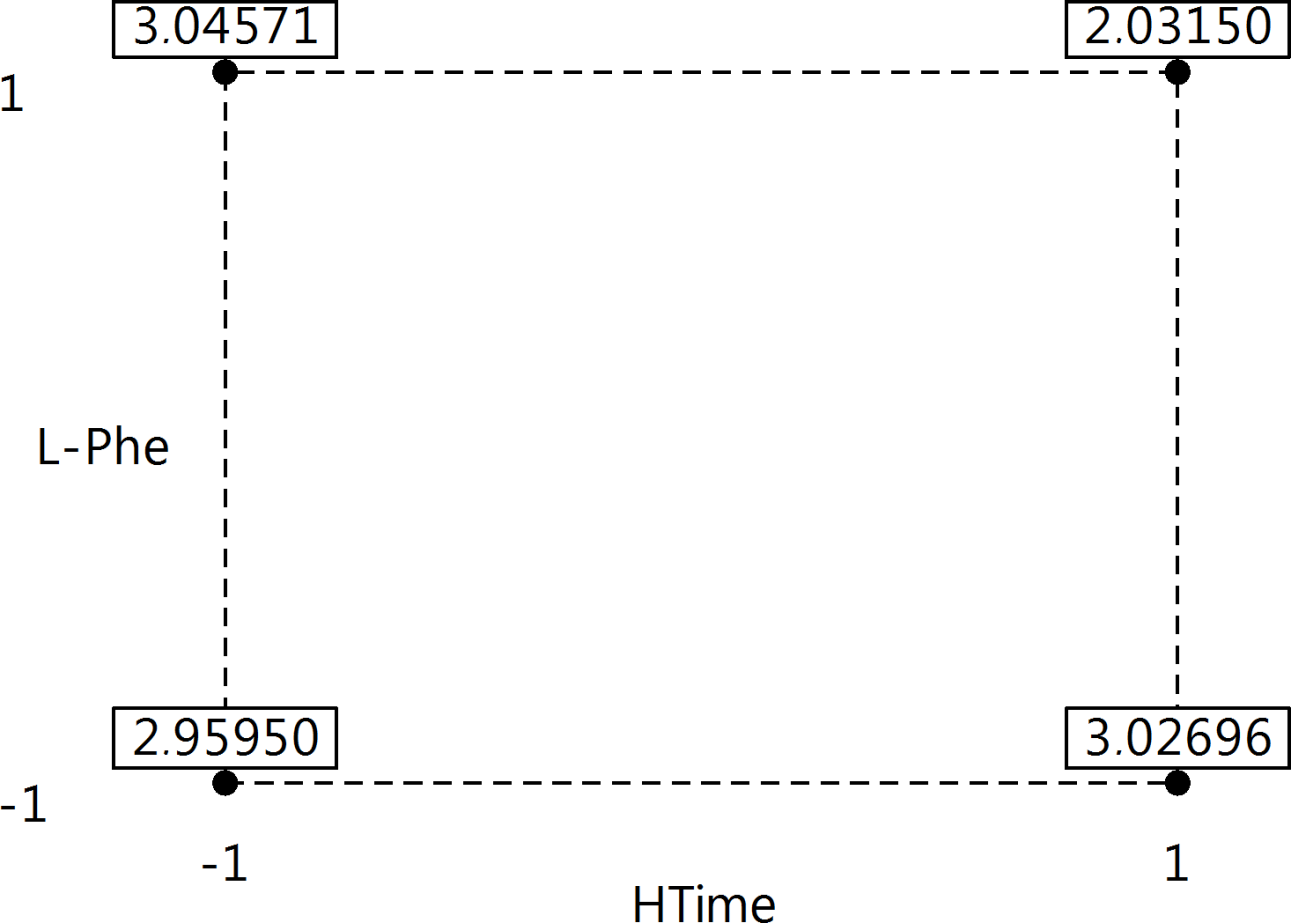
The predicted dry weight for four treatments of significant factors HTime and L-Phe. HTime codes: -1 = 2^nd^ day, +1 = 8^th^ day; L-Phe codes: -1 = lack of L-Phe, +1 = 2.4 g/L of L-Phe.

The final model predicted that maximum dry weight was obtained for HTime = -1 and L-Phe = +1 (Fig 13), which means, in the real world, the harvesting time on the 2^nd^ day and the concentration of L Phe 2.4 g/L. The dry weight predicted for such settings was 3.05 with 95% confidence interval (2.75; 3.34).

#### Harpagide

**Fig 14:**
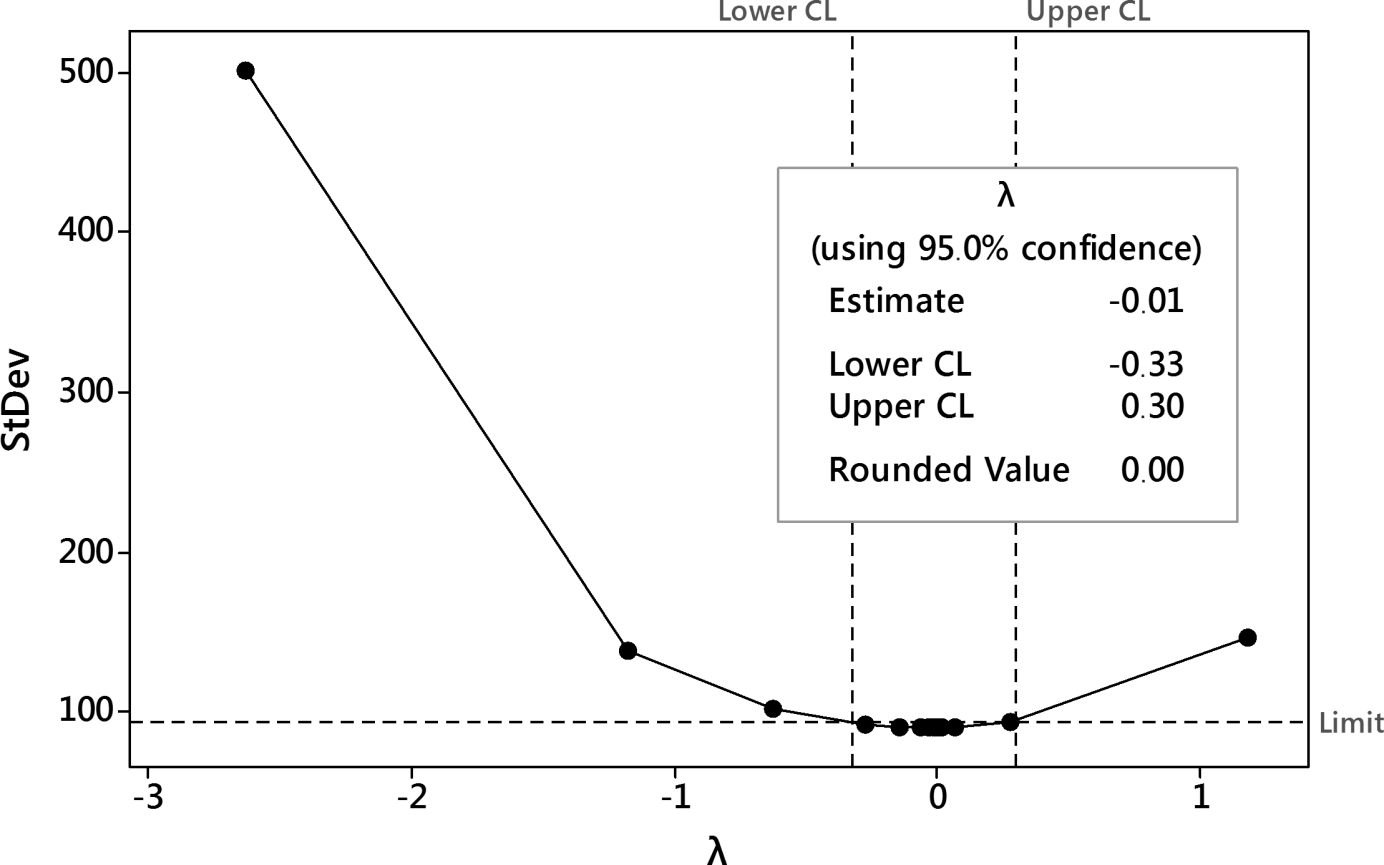
The Box-Cox transformation plot of harpagide outcome. The selected best value of λ parameter was 0 i.e. logarithmic transformation was selected.

The first-step analysis of dataset reported strong heteroscedasticity of data and the Box-Cox procedure was performed (Fig 14). After data processing, the transformation with the parameter λ = 0 was selected as best-fitted i.e. natural logarithm transformation of the harpagide outcome.

**Fig 15:**
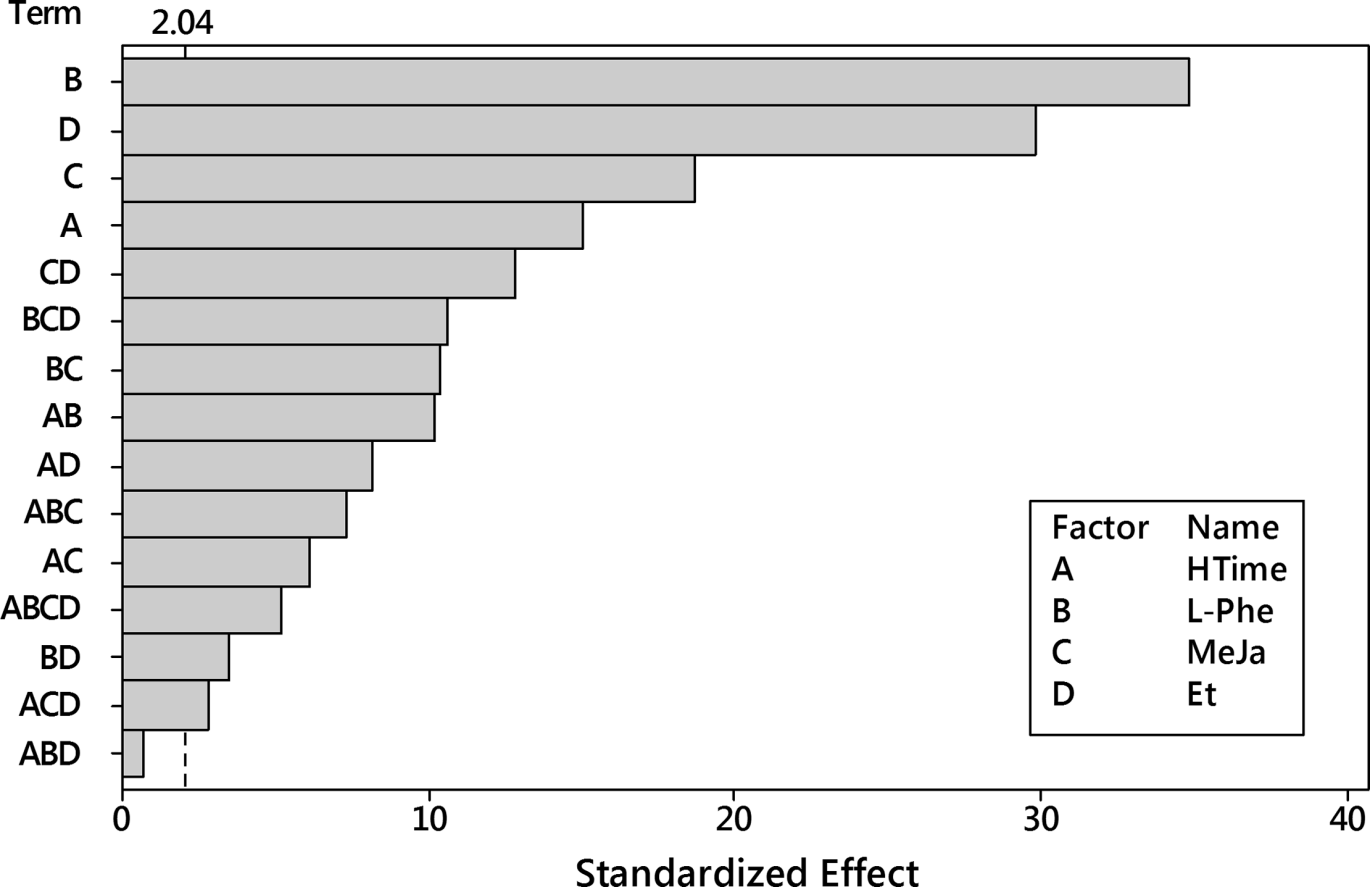
Pareto plot of standardized effects for the transformed harpagide outcome. Four factors (HTime, L-Phe, MeJa, Eth) and their interaction up to the 4^th^ order were considered. Significance level α = 0.05, t = 2.042.

Next, the ANOVA analysis was performed for the logarithm of harpagide with the 4^th^-order model. It reported insignificance for the term of the three-way interaction HTime x L-Phe x Eth (Fig 15).

**Fig 16:**
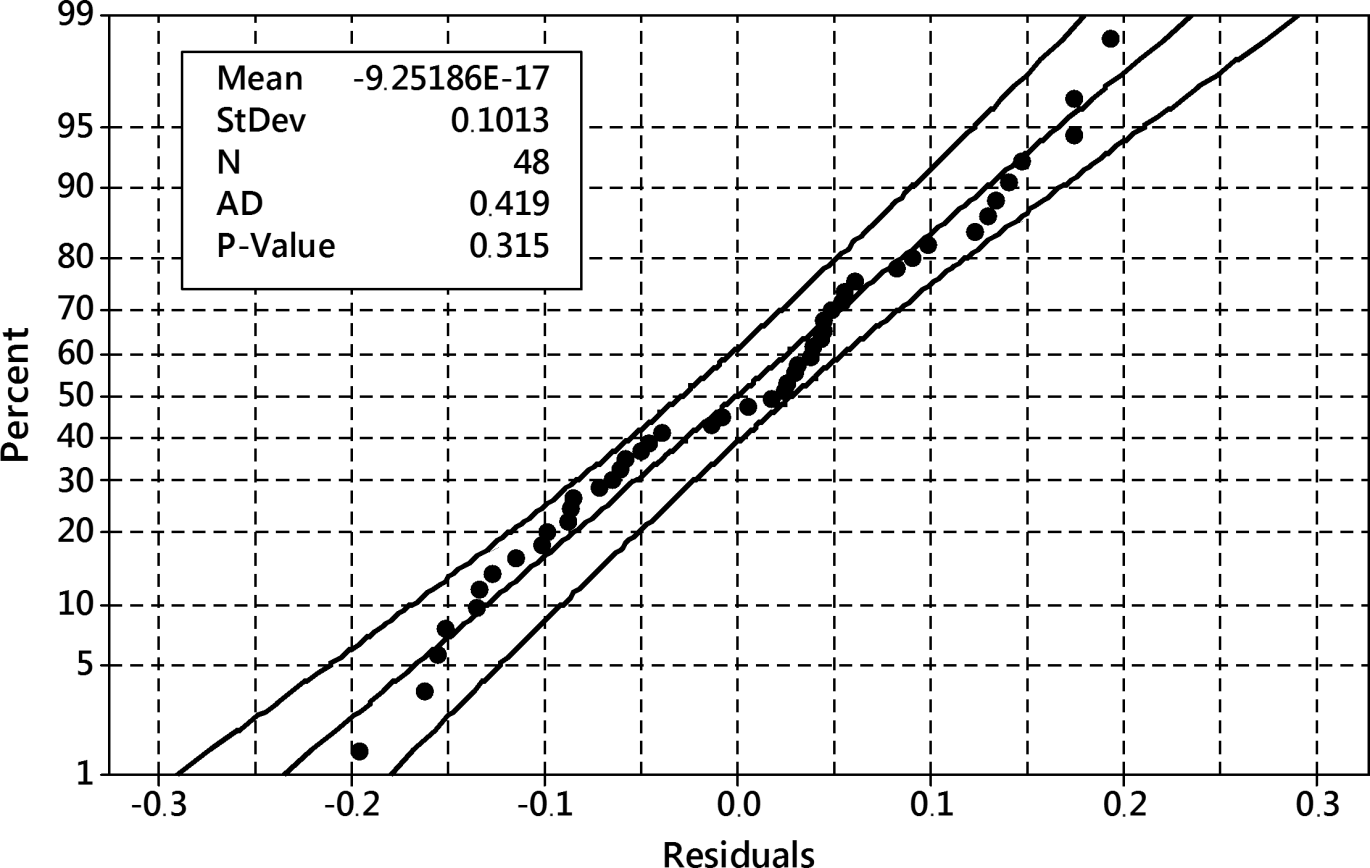
The probability plot of residuals for the reduced model of the transformed harpagide outcome. 95% confidence band, Anderson-Darling test of normality p = 0.315.

After removing this term, the ANOVA analysis was performed again and it reported a well-fitted model with residuals (Fig 16) following the normal distribution–they passed Anderson-Darling’s normality test with p = 0.315.

Next, Tukey’s HSD test was performed and it revealed 10 homogeneous groups inside the dataset (Table 9).

**Table 9:** Homogeneous groups inside the harpagide dataset detected by Tukey’s HSD test.

HTime	L-Phe	MeJa	Eth	Ln(Hp)	1	2	3	4	5	6	7	8	9	10
-1	-1	-1	-1	3.17	*									
1	-1	-1	-1	3.22	*	*								
-1	-1	-1	1	3.54		*								
-1	1	-1	-1	3.88			*							
1	-1	1	-1	4.03			*	*						
-1	-1	1	-1	4.33				*	*					
1	1	-1	-1	4.47					*					
1	-1	-1	1	4.65					*	*				
1	-1	1	1	4.82						*	*			
-1	1	1	1	4.91						*	*			
-1	1	1	-1	4.91						*	*			
-1	-1	1	1	5.06							*			
1	1	1	-1	5.44								*		
-1	1	-1	1	5.51								*		
1	1	1	1	6.06									*	
1	1	-1	1	6.42										*

The maximum harpagide outcome was predicted undoubtedly for HTime = +1, L-Phe = +1, MeJa = -1 and Eth = +1 (see Table 9, last raw), which means, in the real world, the harvesting time on the 2^nd^ day, the concentration of L-Phe 2.4 g/L, a lack of MeJa and the supplementation with the ethephon. The harpagide outcome predicted for such settings was 596 mg/100 g DW with 95% confidence interval (523; 679).

#### 8-*O*-acetyl-harpagide

**Fig 17:**
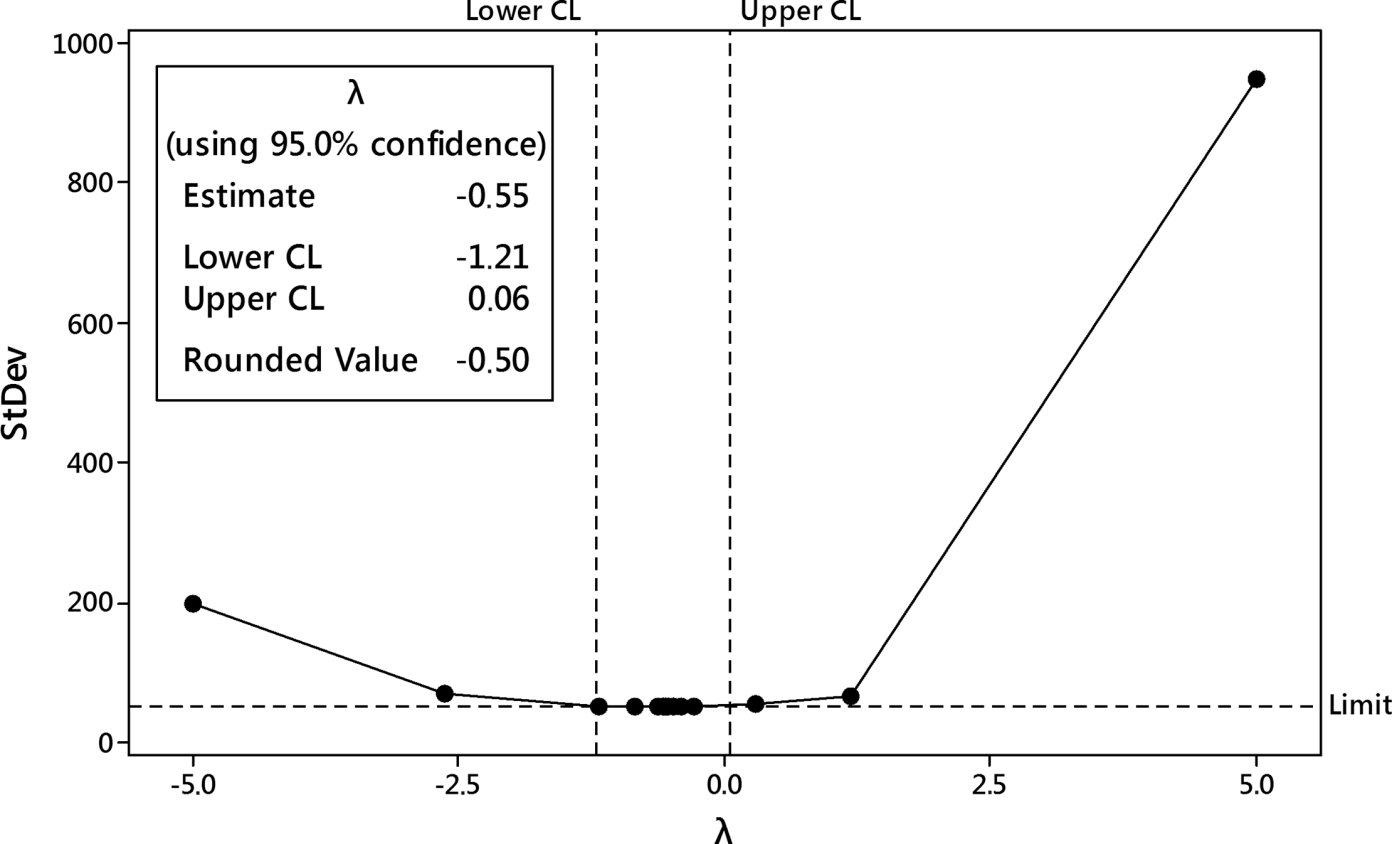
The Box-Cox transformation plot of 8-*O*-acetyl-harpagide outcome. The selected best values of λ parameter is -0.5 i.e. reciprocal square root transformation is selected.

The first-step analysis of dataset reported strong heteroscedasticity of data and the Box-Cox procedure was performed (Fig 17). After data processing, the transformation with the parameter λ = -0.5 was selected as best-fitted i.e. reciprocal of square root of the 8-*O*-acetyl-harpagide outcome.

**Fig 18:**
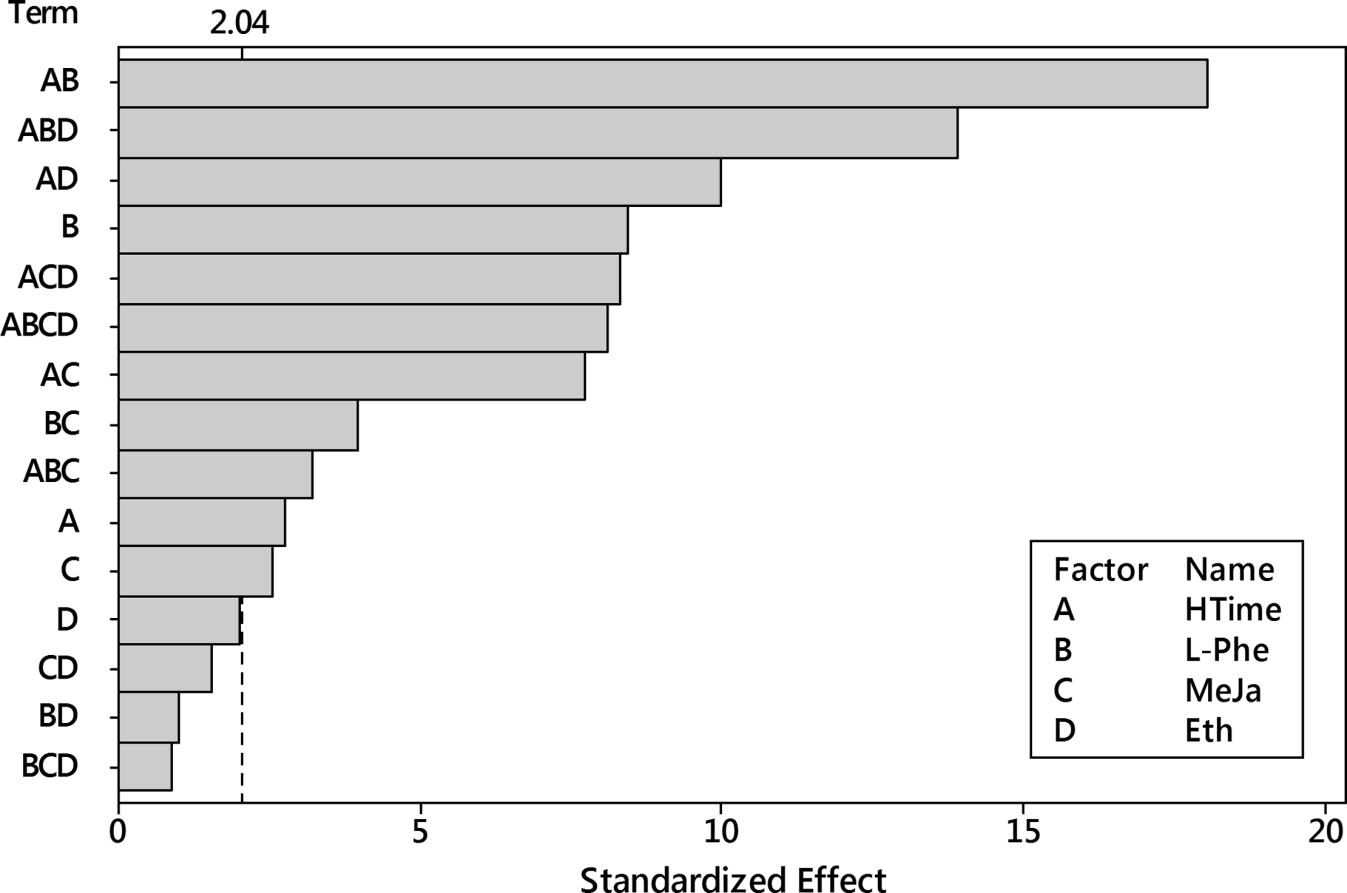
Pareto plot of standardized effects for the transformed 8-*O*-acetyl-harpagide outcome. Four factors (HTime, L-Phe, MeJa, Eth) and their interaction up to 4^th^ order were considered. Significance level α = 0.05, t = 2.042.

Next, the ANOVA analysis was performed for the transformed dataset with the 4^th^-order model. It reported insignificance for four terms: one linear effect (ethephon), two two-way interactions (L-Phe x Eth, MeJa x Eth) and one three-way interaction (L-Phe x MeJa x Eth) (Fig 18).

**Fig 19:**
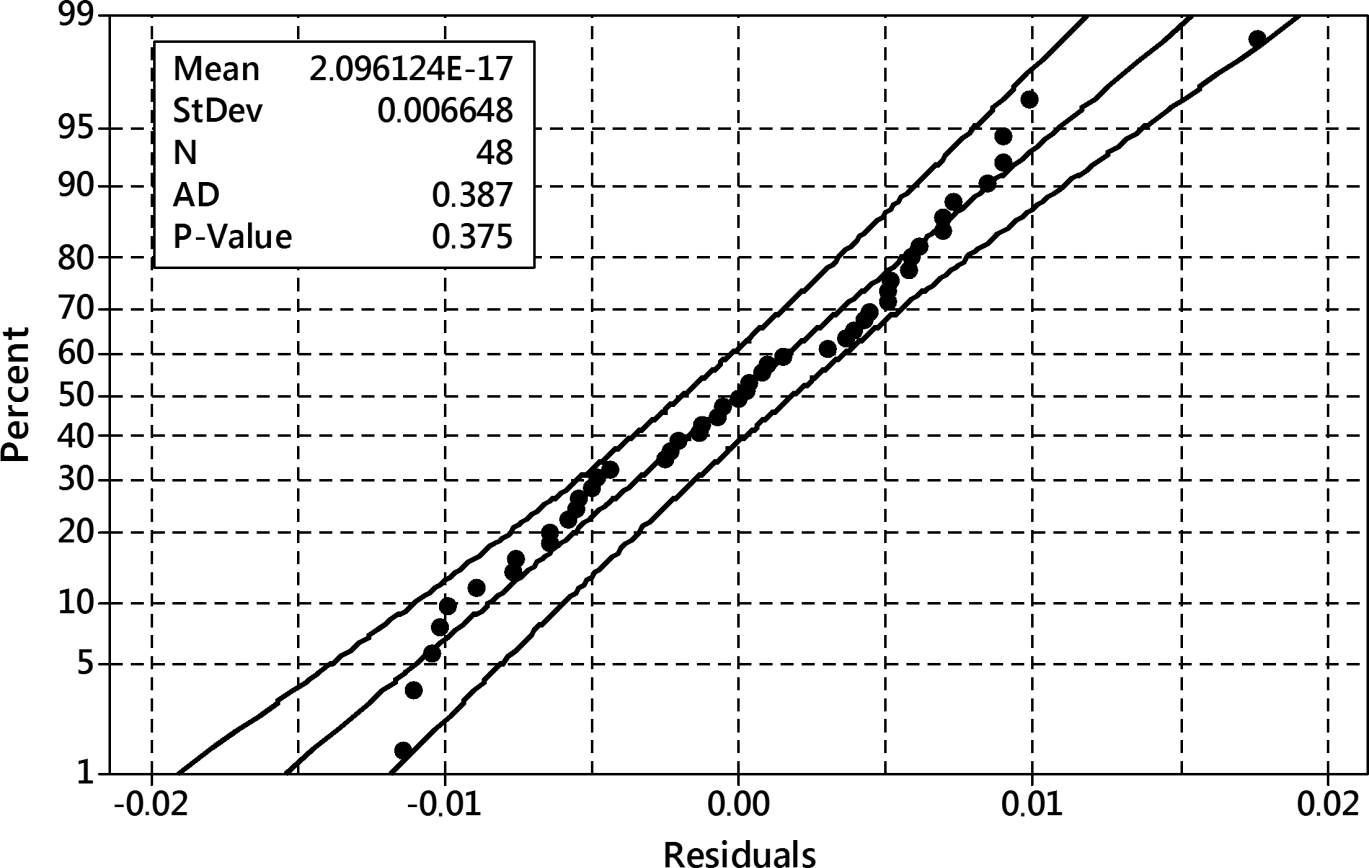
The probability plot of residuals for the reduced model of the transformed 8-*O*-acetyl-harpagide outcome. 95% confidence band, Anderson-Darling test of normality p = 0.375.

After removing these terms, the ANOVA analysis was performed again and it reported a well-fitted model with residuals (Fig 19) following the normal distribution–they passed Anderson-Darling’s normality test with p = 0.375.

Next, Tukey’s HSD test was performed and it revealed 5 homogeneous groups inside the dataset (Table 10). The maximum of 8-*O*-acetyl-harpagide was, because of the reciprocal transformation, located at the minimum of transformed outcome associated with the first homogenous group (Table 10). The optimum conditions for the maximum of 8-*O*-acetyl-harpagide were HTime = +1, L-Phe = +1, Eth = +1 while the setting of MeJa was not significant (both settings of MeJa were located inside the same homogeneous group).

**Table 10:** Homogeneous groups inside the transformed 8-*O*-acetyl-harpagide dataset detected by Tukey’s HSD test. Box-Cox transformation is for λ = -0.5 i.e. reciprocal of square root of the 8-*O*-acetyl-harpagide outcome.

HTime	L-Phe	MeJa	Eth	BoxCox	1	2	3	4	5
1	1	1	1	0.062860	*				
1	1	-1	1	0.079691	*	*			
-1	1	1	-1	0.085445		*			
1	1	-1	-1	0.093875		*			
-1	-1	1	1	0.095990		*			
-1	-1	1	-1	0.100235		*			
-1	-1	-1	1	0.130378			*		
-1	1	-1	-1	0.130998			*		
1	1	1	-1	0.135358			*		
-1	-1	-1	-1	0.138409			*	*	
1	-1	-1	-1	0.138653			*	*	
1	-1	1	-1	0.147035			*	*	
-1	1	-1	1	0.147808			*	*	
1	-1	-1	1	0.148045			*	*	
1	-1	1	1	0.158273				*	
-1	1	1	1	0.180459					*

It means, in the real world, the harvesting time on the 8^th^ day, the concentration of L-Phe 2.4 g/L and the supplementation with the ethephon 50 μM. The maximum 8-*O*-acetyl-harpagide outcome predicted for all settings on +1 (with MeJa supplementation) was 305 mg/100 g DW with 95% confidence interval (237; 407).

#### Total of harpagide and 8-*O*-acetyl-harpagide

**Fig 20:**
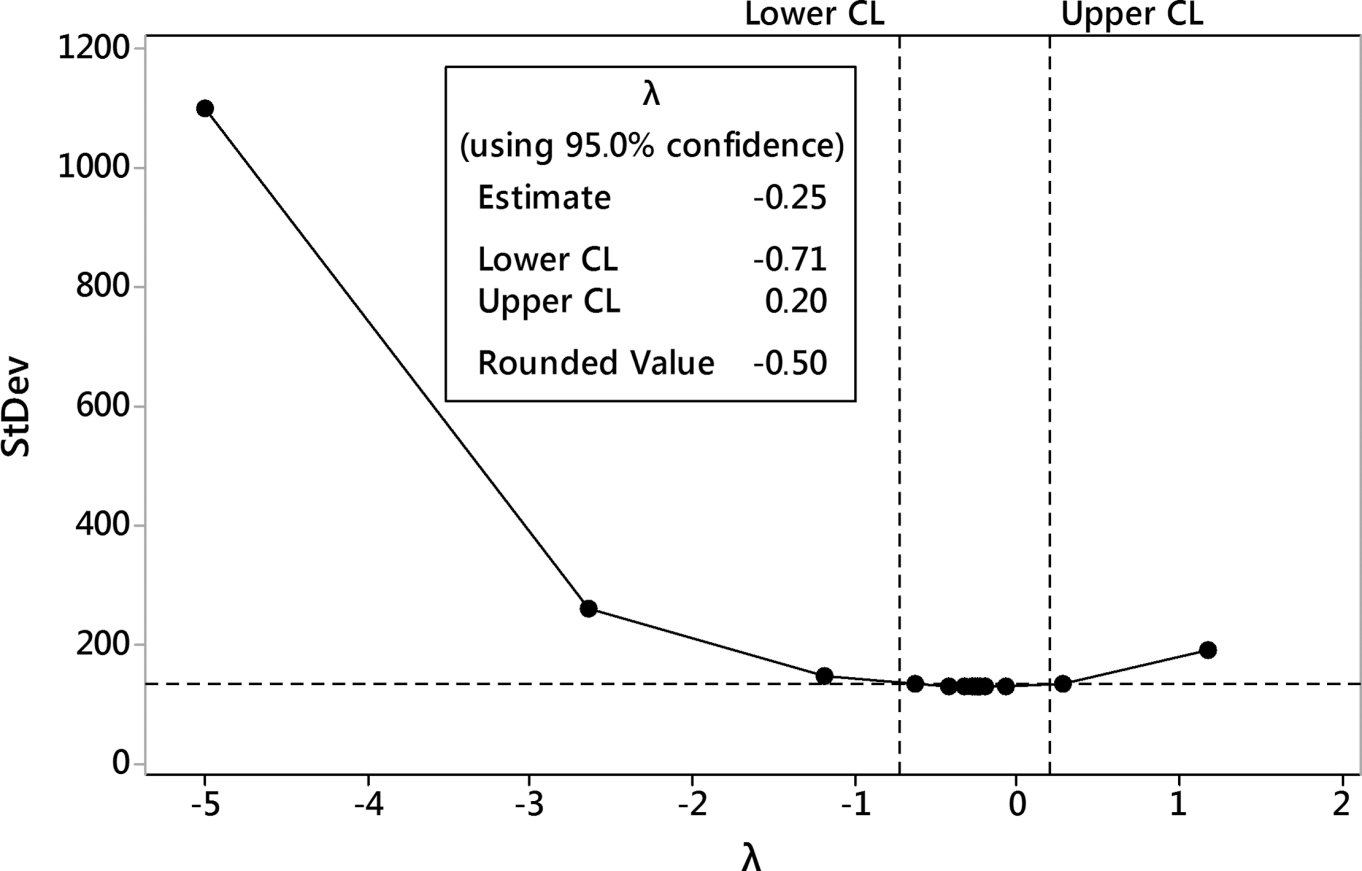
Box-Cox transformation plot of the total of harpagide and 8-*O*-acetyl-harpagide outcome. **The** The selected best values of λ parameter was -0.5 i.e. reciprocal of square root transformation was selected.

The first-step analysis of the dataset reported strong heteroscedasticity of the data and the Box-Cox procedure was performed (Fig 20). After data processing, the transformation with the parameter λ = -0.5 was selected as best-fitted i.e. reciprocal of square root of the total of harpagide and 8-*O*-acetyl-harpagide outcome.

**Fig 21:**
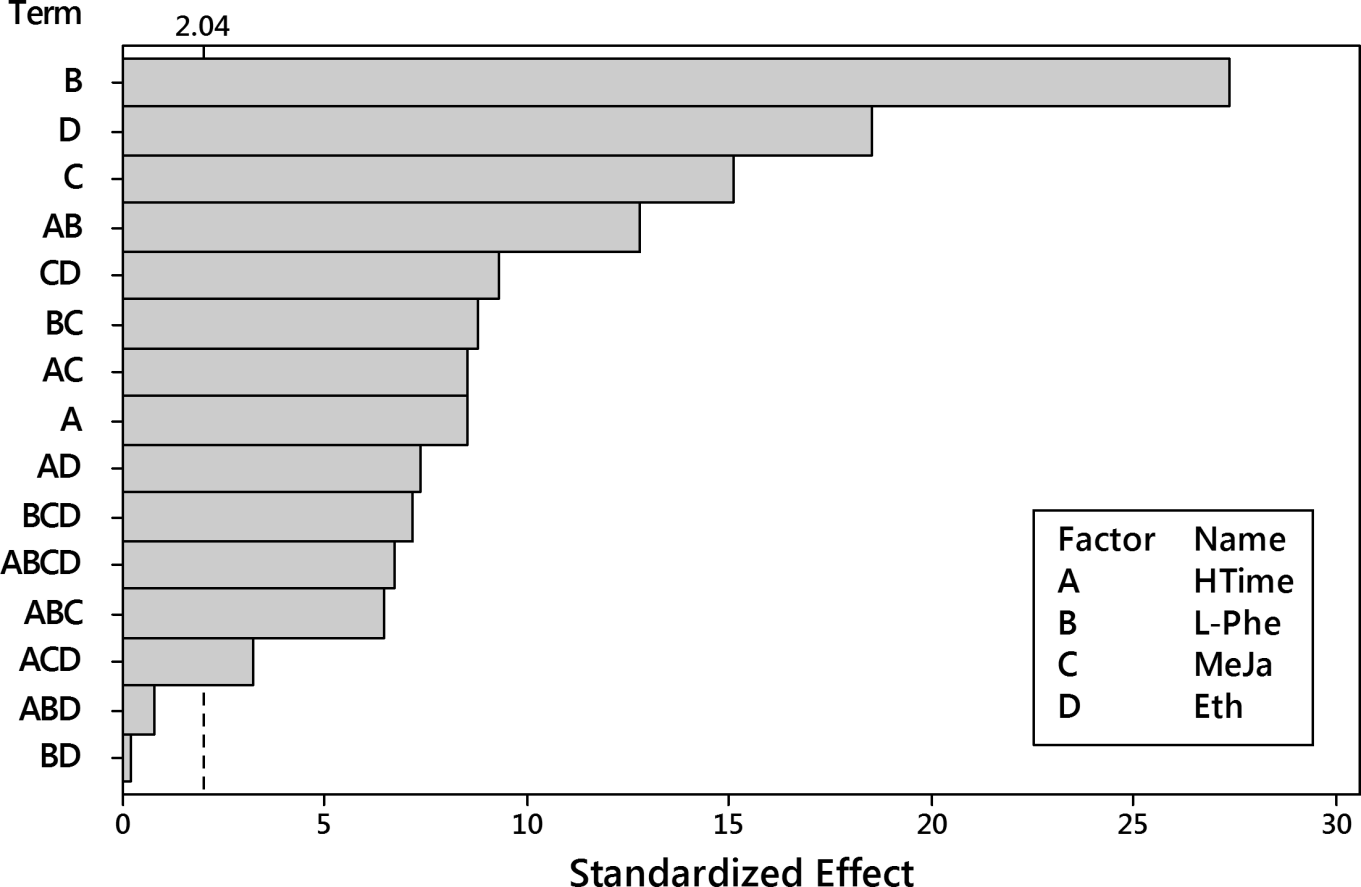
Pareto plot of standardized effects for the transformed total of harpagide and 8-*O*-acetyl-harpagide outcome. Four factors (HTime, L-Phe, MeJa, Eth) and their interaction up to the 4^th^ order were considered. Significance level α = 0.05, t = 2.042.

Next, the ANOVA analysis was performed for the transformed dataset with the 4^th^-order model. It reported insignificance for two terms: two-way interaction (L-Phe x Eth) and one three-way interaction (HTime x L-Phe x Eth) (Fig 21).

**Fig 22:**
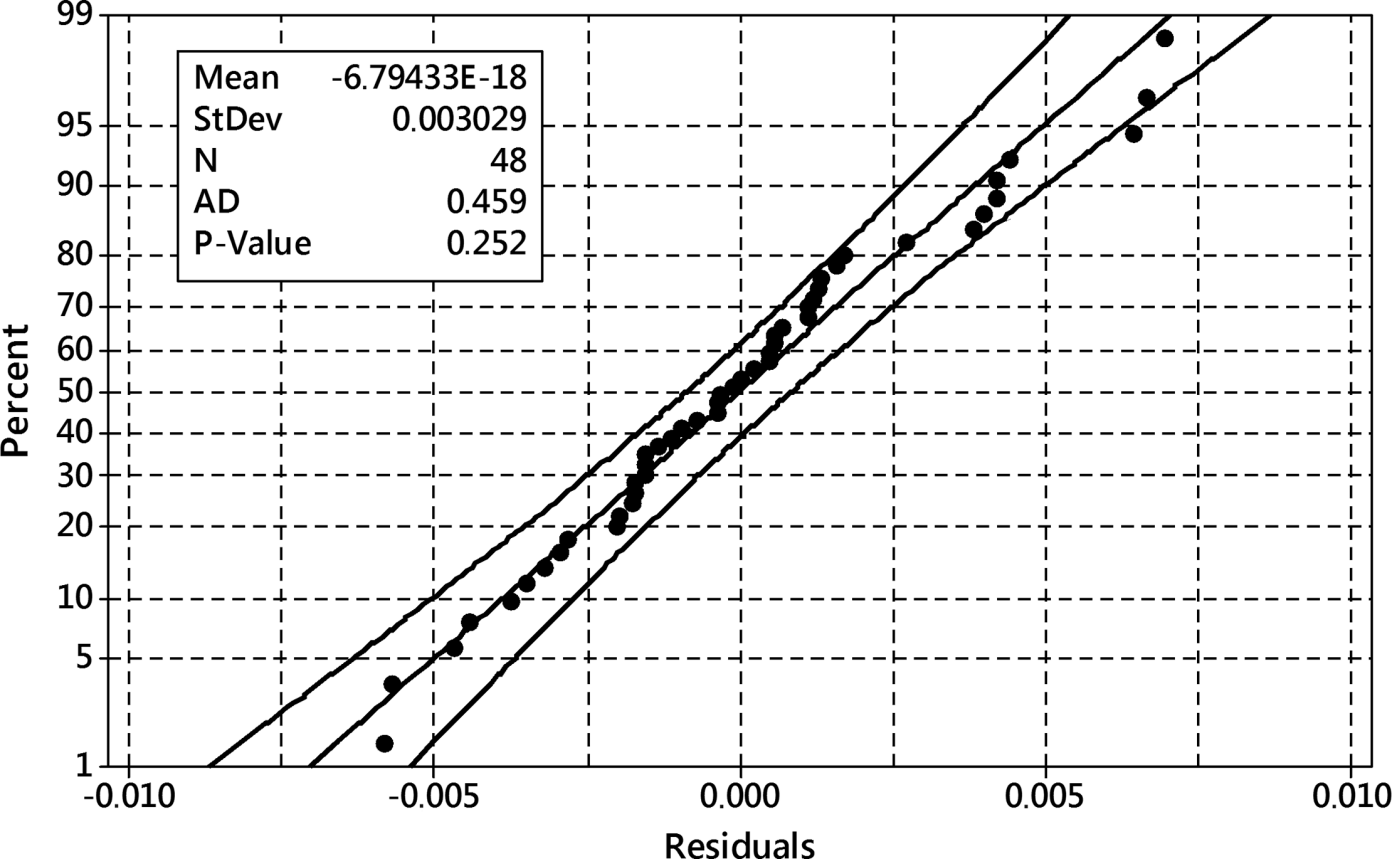
The probability plot of residuals for the reduced model of the transformed total of iridoids outcome. 95% confidence band, Anderson-Darling test of normality p = 0.252.

After removing these terms, the ANOVA analysis was performed again and it reported a well-fitted model with residuals (Fig 22) following the normal distribution–they passed Anderson-Darling’s normality test with p = 0.252.

Next, Tukey’s HSD test was performed and it revealed 8 homogeneous groups inside the dataset (Table 11). The maximum of the total of harpagide and 8-*O*-acetyl-harpagide was, because of the reciprocal transformation, located at the minimum of transformed outcome associated with the first homogenous group (Table 11).

**Table 11:** Homogeneous groups inside the transformed total of harpagide and 8-*O*-acetyl-harpagide dataset detected by Tukey’s HSD test. Box-Cox transformation is for λ = -0.5 i.e. reciprocal of square root of the total of harpagide and 8-*O*-acetyl-harpagide outcome.

HTime	L-Phe	MeJa	Eth	BoxCox	1	2	3	4	5	6	7	8
1	1	-1	1	0.035941	*							
1	1	1	1	0.038307	*							
-1	1	-1	1	0.058528		*						
1	1	1	-1	0.059229		*						
-1	1	1	-1	0.060353		*	*					
-1	-1	1	1	0.061158		*	*					
1	1	-1	-1	0.070507			*	*				
-1	-1	1	-1	0.075356				*	*			
-1	1	1	1	0.077363				*	*			
1	-1	1	1	0.078059				*	*			
1	-1	-1	1	0.081615					*			
-1	1	-1	-1	0.096867						*		
1	-1	1	-1	0.098774						*		
-1	-1	-1	1	0.103309						*	*	
1	-1	-1	-1	0.113929							*	*
-1	-1	-1	-1	0.114666								*

The optimum conditions for the maximum of 8-*O*-acetyl-harpagide were HTime = +1, L-Phe = +1, Eth = +1 while the setting of MeJa was not significant (both settings of MeJa were located inside the same homogeneous group). It means, in the real world, the harvesting time on the 8^th^ day, the concentration of L-Phe 2.4 g/L and the supplementation with the ethephon 50 μM. The maximum of the total of harpagide and 8-*O*-acetyl-harpagide outcome predicted for all settings on +1 (with MeJa supplementation) was 761 mg/100 g DW with 95% confidence interval (620; 956).

#### Total productivity of the process (harpagide and 8-*O*-acetyl-harpagide per flask)

**Fig 23:**
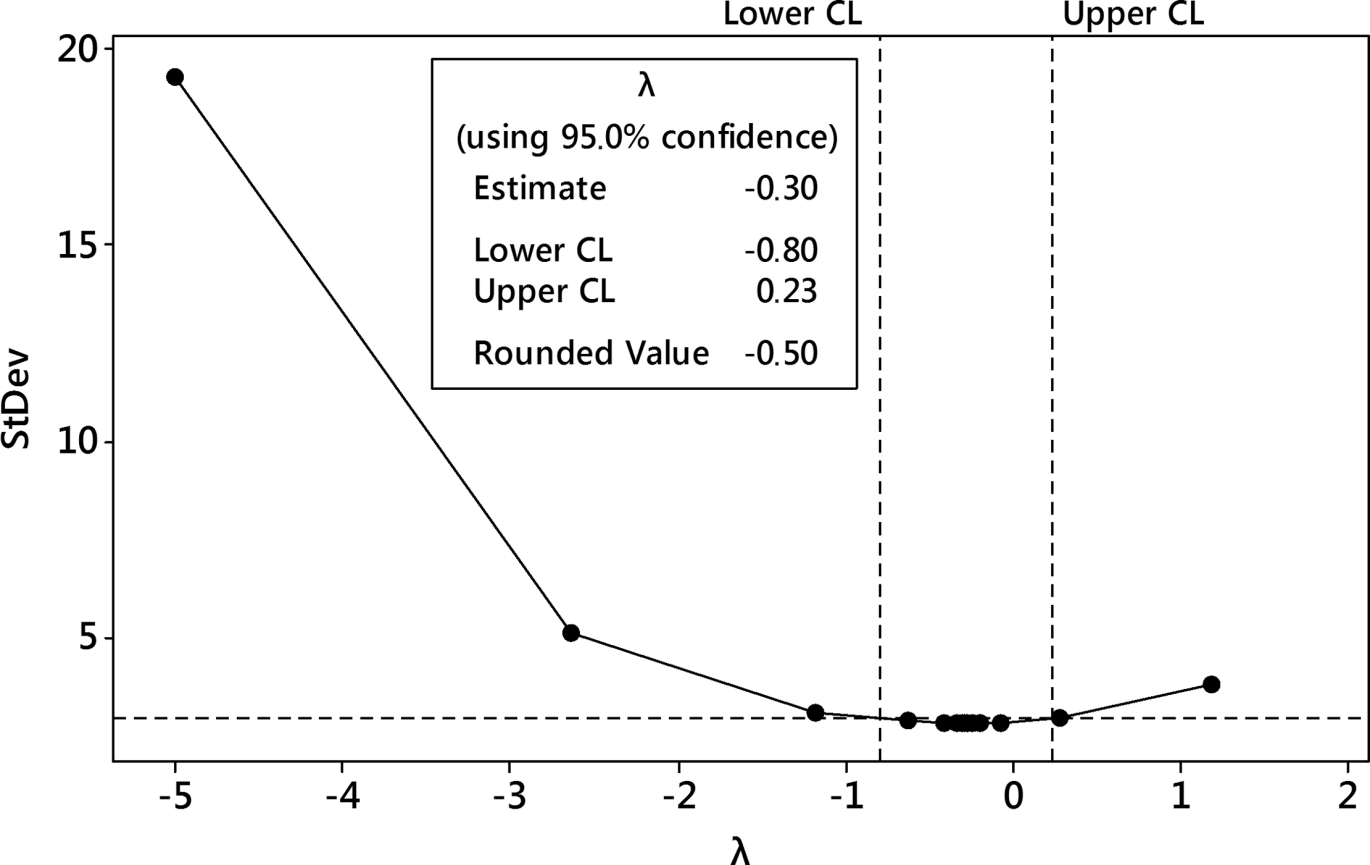
The Box-Cox transformation plot of the total productivity of the process (iridoids outcome per flask). The selected best values of λ parameter was -0.5 i.e. reciprocal of square root transformation was selected.

The first-step analysis of the dataset reported strong heteroscedasticity of the data and the Box-Cox procedure was performed (Fig 23). After data processing, the transformation with the parameter λ = -0.5 was selected as best-fitted i.e. reciprocal of square root of the total productivity per flask i.e. harpagide and 8-*O*-acetyl-harpagide outcome per flask.

**Fig 24:**
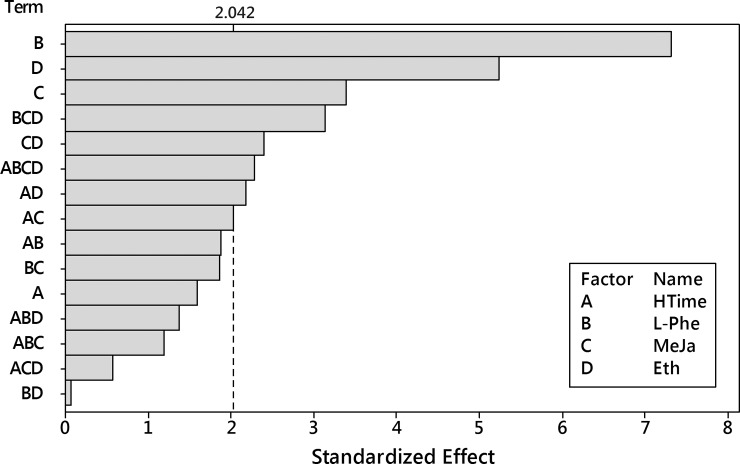
Pareto plot of standardized effects for the transformed total productivity of the process (iridoids outcome per flask). Four factors (HTime, L-Phe, MeJa, Eth) and their interaction up to the 4^th^ order were considered. Significance level α = 0.05, t = 2.042.

Next, the ANOVA analysis was performed for the transformed dataset with the 4^th^-order model. It reported insignificance for two terms: one main effect, four two-way interaction and three three-way interaction (Fig 24).

**Fig 25:**
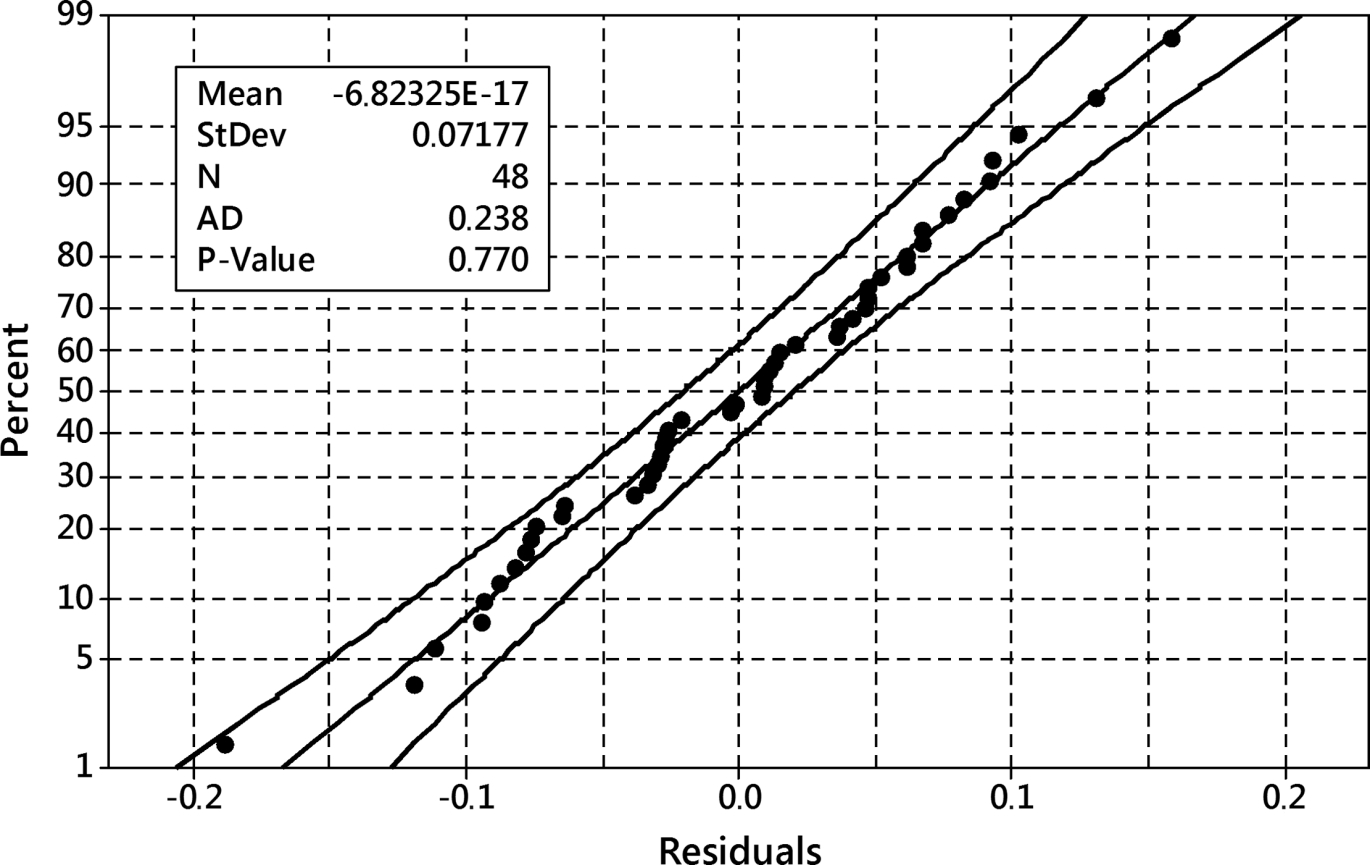
The probability plot of residuals for the reduced model of the transformed total productivity of the process. Productivity defined as iridoids outcome per flask. 95% confidence band, Anderson-Darling test of normality p = 0.770.

After removing these terms, the ANOVA analysis was performed again and it reported a well-fitted model with residuals (Fig 25) following the normal distribution–they passed Anderson-Darling’s normality test with p = 0.770.

Next, Tukey’s HSD test was performed and it revealed 7 homogeneous groups inside the dataset (Table 12). The maximum of the total productivity per flask was, because of the reciprocal transformation, located at the minimum of transformed outcome associated with the first homogenous group (Table 12).

**Table 12:** Homogeneous groups inside the transformed total productivity (harpagide and 8-*O*-acetyl-harpagide per flask) dataset detected by Tukey’s HSD test. Box-Cox transformation is for λ = -0.5 i.e. reciprocal of square root of the total productivity outcome.

HTime	L-Phe	MeJa	Eth	BoxCox	1	2	3	4	5	6	7
1	1	-1	1	0.245212	*						
1	1	1	1	0.285898	*	*					
-1	1	1	-1	0.334577	*	*	*				
-1	1	-1	1	0.346931	*	*	*	*			
-1	-1	1	1	0.380956	*	*	*	*	*		
1	1	1	-1	0.411229	*	*	*	*	*	*	
-1	1	1	1	0.468675		*	*	*	*	*	*
1	-1	1	1	0.470346		*	*	*	*	*	*
1	1	-1	-1	0.479100		*	*	*	*	*	*
1	-1	-1	1	0.491751		*	*	*	*	*	*
-1	-1	1	-1	0.518817			*	*	*	*	*
-1	1	-1	-1	0.552087				*	*	*	*
1	-1	1	-1	0.571391					*	*	*
-1	-1	-1	1	0.602040						*	*
1	-1	-1	-1	0.632668							*
-1	-1	-1	-1	0.640717							*

It means that six different treatments constitute possible optimum settings statistically proved however the greatest prediction of the total productivity was HTime = +1, L-Phe = +1, Eth = +1 with the lack of MeJa. It means, in real world, the harvesting time on the 8^th^ day, the concentration of L-Phe 2.4 g/L and the supplementation with the ethephon 50 μM. The predicted maximum of the total productivity per flask was 9.96 mg/flask with 95% confidence interval (6.89; 15.68) while three experimentally observed values for this treatment were 12.41, 19.09, 20.12 mg/flask.

## Conclusions

To the best of our knowledge, it is the first time that the analysis of harpagide and 8-*O*-acetyl-harpagide in *M*. *melissophyllum* shoot cultures has been reported. We can conclude that elicitation with MeJa and Eth, and medium supplementation with L-Phe significantly increased the accumulation of harpagide and 8-*O*-acetyl-harpagide in *M*. *melissophyllum* agitated shoot cultures. The highest amounts of iridoids were detected on the 8^th^ day after medium supplementation: harpagide 0.62% DW (variant F: L-Phe 2.4 g/L; Eth 50 μM), 8-*O*-acetyl-harpagide 0.26% DW (variant H: L-Phe 2.4 g/L; Eth 50 μM; MeJa 50 μM) and the total amount of the two iridoids 0.78% DW (variant F). The results indicate that *M*. *melissophyllum* agitated shoot cultures might be considered as a potential biotechnological source of harpagide and 8-*O*-acetyl-harpagide.

## Supporting information

S1 Dataset The experimental design scheme and the raw dataset. (XLSX)
